# SLC11A1 protein as a key regulator of iron metabolism, ferroptosis mediator, and putative therapeutic target in nonalcoholic fatty liver disease: an integrated bioinformatics analysis

**DOI:** 10.3389/fphar.2025.1715699

**Published:** 2025-11-25

**Authors:** Yang Wang, Bugao Zhou, Shanshan Li, Linxin Zheng, Xiongfeng Huang, Huiyu Wang, Sili Li, Yuhan Lin, Yanhe Xu

**Affiliations:** 1 Department of Postgraduate, Jiangxi University of Chinese Medicine, Nanchang, China; 2 Scientific Research Department of Jiangxi University of Chinese Medicine, College of Traditional Chinese Medicine, Jiangxi University of Chinese Medicine, Key Laboratory of Formula-pattern research, Jiangxi Provincial Engineering Research Center of Development and Evaluation of TCM classic prescriptions, Key Laboratory of Prevention and Treatment of Immunological and Metabolic Diseases Related to Prescription and Syndrome, Nanchang, China; 3 Laboratory Animal Science and Technology Centre, Jiangxi University of Chinese Medicine, Nanchang, China

**Keywords:** nonalcoholic fatty liver disease, integrated bioinformatics analysis, hub genes, iron metabolism, ferroptosis

## Abstract

**Background:**

Nonalcoholic fatty liver disease (NAFLD) has become one of the most prevalent chronic liver diseases worldwide, with its incidence closely linked to metabolic syndromes such as obesity and diabetes. Studies have indicated that dysregulated iron metabolism and ferroptosis play critical roles in its pathological progression, underscoring the urgent need for in-depth exploration of novel biomarkers and therapeutic strategies.

**Methods:**

This study utilized NAFLD datasets from the GEO database and applied bioinformatics approaches to identify iron metabolism and ferroptosis-related differentially expressed genes (DEGs) in NAFLD. Key regulatory proteins—ERN1, SLC11A1, MYC, TLR7, and PPARGC1A—were screened using weighted gene co-expression network analysis (WGCNA) and a machine learning algorithm (LASSO). Their correlations with immune microenvironment features were also evaluated. Validation sets confirmed the differential expression of ERN1 and SLC11A1, with area under the receiver operating characteristic curve (AUC) values of 0.855 and 0.89, respectively, and a combined AUC of 0.923. Additionally, single-cell RNA sequencing (scRNA-seq) was applied to analyze the cell type-specific expression and functional characteristics of these genes during NAFLD development. Molecular docking coupled with molecular dynamics simulations was employed to evaluate the binding patterns and dynamic stability of Resmetirom—a drug approved for the treatment of nonalcoholic fatty liver disease in adults—with the protein structures of ERN1 and SLC11A1. Finally, quantitative reverse transcription polymerase chain reaction (qRT-PCR) was used to validate the expression differences of key protein biomarkers at the tissue level.

**Results:**

A total of 26 iron metabolism/ferroptosis-related DEGs significantly associated with NAFLD were identified. Machine learning algorithms confirmed ERN1, SLC11A1, MYC, TLR7, and PPARGC1A as diagnostic biomarkers. Immune microenvironment analysis elucidated correlations between the expression of these key proteins and immune cell infiltration. Molecular docking and dynamics simulations predicted that Resmetirom may exert a potential targeted effect by stably binding to the protein structures of ERN1 and SLC11A1. Experimental validation confirmed significant differential expression of ERN1 and SLC11A1 proteins in NAFLD tissues.

**Conclusion:**

This study successfully identified specific proteins related to iron metabolism/ferroptosis pathways, such as ERN1 and SLC11A1, which demonstrate significant diagnostic potential for NAFLD, with SLC11A1 potentially possessing greater diagnostic value as a biomarker. The findings enhance the understanding of the genetically regulated pathogenesis of NAFLD and provide an important foundation for developing innovative diagnostic approaches and therapeutic interventions based on these targets.

## 1 Introduction

Nonalcoholic fatty liver disease (NAFLD) ranks among the most prevalent chronic liver disorders worldwide, affecting approximately one-quarter of the global adult population and markedly increasing the risk of cirrhosis, hepatic failure, and hepatocellular carcinoma (Ching-Yeung et al., 2019; Ipsen et al., 2018). The core pathophysiology of NAFLD centers on the dysregulation of hepatic lipid metabolism, defined by the excessive accumulation of lipids—predominantly triglycerides—within hepatocytes. This lipid overload triggers oxidative stress, inflammatory responses, hepatocyte injury, and progressive liver fibrosis (Castillo et al., 2021). Furthermore, hypoxia, aberrant angiogenesis, and immune dysregulation—such as complement system activation—collectively contribute to disease progression (Schwabe et al., 2020; Wiering et al., 2023). Although early-stage NAFLD (e.g., simple steatosis) can be effectively alleviated via lifestyle interventions—including dietary adjustments, weight management, and regular physical exercise, all of which have been shown to markedly reduce hepatic inflammation and steatosis—approved pharmacotherapies remain scarce. Significant challenges persist in predicting and intervening in the progression of NAFLD to nonalcoholic steatohepatitis (NASH) and liver fibrosis (Sheka et al., 2020). These therapeutic gaps highlight an urgent need to conduct in-depth research into the molecular mechanisms underlying NAFLD, with the goal of developing precise and effective treatment strategies to ultimately lower its associated morbidity and mortality.

Molecular biomarkers play a pivotal role in elucidating the pathogenesis of NAFLD, facilitating noninvasive disease staging (e.g., differentiating simple steatosis from NASH or liver fibrosis), and monitoring responses to therapy. Circulating microRNAs (miRNAs), long non-coding RNAs (lncRNAs), and recently discovered genes such as GADD45G and NUPR1 exhibit considerable potential in unraveling relevant molecular pathways, predicting disease progression, and guiding personalized therapeutic strategies (Sun and Kemper, 2023; Jiang et al., 2024). Nevertheless, the clinical translation of these biomarkers is impeded by an incomplete understanding of their roles within complex molecular networks, a paucity of large-scale prospective validation studies, and interindividual heterogeneity in their expression levels. Thus, additional research is needed to identify and validate novel biomarkers, as well as to explore patterns of their combinatorial application. These advancements would improve diagnostic accuracy and specificity, optimize therapeutic decision-making, and ultimately alleviate the substantial health and economic burdens posed by NAFLD.

Iron metabolism plays a pivotal role in energy production, heme biosynthesis, and DNA repair, as it sustains iron ion homeostasis and redox balance in the body. Under pathological conditions—such as excessive lipid overload or insulin resistance—disturbed hepatocyte homeostasis triggers iron dysregulation, which is manifested by hepatic iron accumulation and excessive generation of reactive oxygen species (ROS); this cascade ultimately activates the ferroptosis signaling pathway (Guan et al., 2023). During the progression of NAFLD, ferroptosis induced by iron overload significantly accelerates the transition from simple steatosis to NASH and subsequent liver fibrosis. This pathological process is mediated primarily via lipid peroxidation-induced cellular damage and mitochondrial dysfunction (Minchenko et al., 2024a). Numerous studies have confirmed aberrant expression of ferroptosis-related markers (e.g., GPX4, ACSL4) in patients with advanced NAFLD. Mechanistically, this ferroptosis-driven pathological progression involves the inhibition of the Nrf2 antioxidant defense pathway and the activation of specific inflammasomes, particularly the NLRP3 inflammasome (Wu et al., 2024a).

This study aimed to systematically explore the regulatory roles of key iron metabolism/ferroptosis-associated genes (ERN1 and SLC11A1) in NAFLD—a globally prevalent metabolic liver disorder—via an integrated multi-omics analytical strategy. First, transcriptome datasets were mined, and machine learning algorithms [least absolute shrinkage and selection operator (LASSO)] combined with weighted gene co-expression network analysis (WGCNA) were applied to screen core target genes, which were subsequently validated as potential diagnostic biomarkers and therapeutic targets for NAFLD. To enhance the reliability of these findings, cross-validation was performed using animal liver tissue samples and single-cell RNA sequencing (scRNA-seq) datasets. Furthermore, molecular docking and molecular dynamics (MD) simulations were utilized to identify potential therapeutic compounds that specifically target these core genes. Collectively, this research not only offers novel insights into the pathogenesis of NAFLD from the perspective of iron metabolism and ferroptosis but also identifies promising molecular candidates to support the development of clinical intervention strategies against NAFLD.

## 2 Methods

### 2.1 Data acquisition and preliminary processing

**FIGURE 1:**
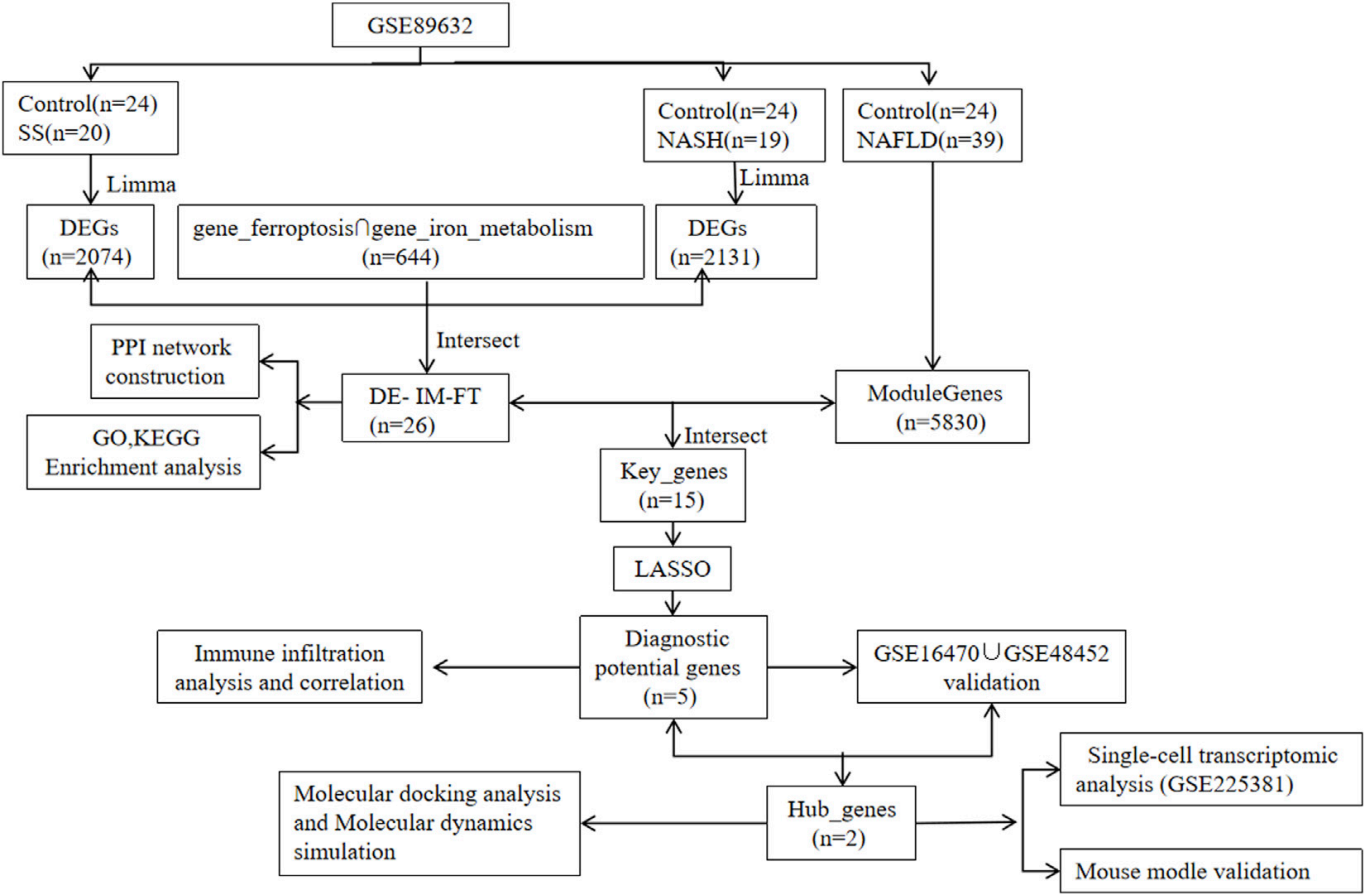
Flow diagram of the analysis process.

The mRNA sequencing datasets GSE89632, GSE16470, and GSE48452 utilized in this study were obtained from the Gene Expression Omnibus (GEO) database under the National Center for Biotechnology Information (NCBI), which were generated from the Illumina HiSeq platform (GPL14951), Affymetrix Human Genome U133 Plus 2.0 Array (GPL13667), and Affymetrix Human Gene 1.0 ST Array (GPL11532) platforms, encompassing normal control (Control), simple steatosis (SS), and nonalcoholic steatohepatitis (NASH) samples; the raw expression data were processed by log2 transformation to achieve normalized distribution, and to define the target gene set associated with iron metabolism and ferroptosis pathways, we integrated and filtered information from the GeneCards and FerrDB V2.0 databases, with the key screening workflow summarized in Figure 1.

### 2.2 Identification of differentially expressed iron metabolism/ferroptosis-related genes

Prior to differential expression analysis, standardized preprocessing of the raw GEO expression matrix was performed using the limma R package, including background correction, cross-sample normalization, and conversion of probe IDs to standard gene symbols; subsequently, based on the preprocessed data, linear model fitting and empirical Bayes tests were conducted with limma to identify differentially expressed genes (DEGs) meeting the criteria of |log_2_FC| > 0.585 and adjusted p-value (adj.P.Val) < 0.05, with results visualized through volcano plots generated by ggplot2 and heatmaps drawn by pheatmap (Stelzer et al., 2016; Li et al., 2024). To identify Iron Metabolism/Ferroptosis (IM-FT)-related DEGs, target gene sets were obtained from the GeneCards database (comprising 1,087 and 1,463 genes for iron metabolism and ferroptosis pathways, respectively) and the FerrDB V2 database (563 ferroptosis-related genes), and Venn diagram analysis was ultimately employed to extract the intersection between DEGs and IM-FT-related genes, yielding the final set of IM-FT-associated differentially expressed genes for subsequent investigation.

### 2.3 Enrichment analysis

A multidimensional functional annotation strategy was implemented whereby Gene Ontology (GO) and Kyoto Encyclopedia of Genes and Genomes (KEGG) enrichment analyses of differentially expressed genes were performed using clusterProfiler, with GO systematically annotating three functional hierarchies—molecular functions (MF; e.g., iron ion binding activity), cellular components (CC; e.g., mitochondrial inner membrane localization), and biological processes (BP; e.g., ferroptosis regulation)—while KEGG elucidated key metabolic and signaling pathways (e.g., ferroptosis pathway hsa04216); all enrichment results underwent Benjamini–Hochberg multiple testing correction with significance thresholds set at p < 0.05 and adjusted p < 0.05; complementary gene set enrichment analysis (GSEA) integrated whole-transcriptome expression rankings to detect coordinated weak-effect gene functions using predefined molecular signatures from the Molecular Signatures Database (MSigDB), employing 5,000 permutations to identify significantly enriched gene sets at FDR q-value <0.25, thereby comprehensively resolving systemic regulatory mechanisms of Iron Metabolism/Ferroptosis (IM-FT)-related functional modules (Yu et al., 2012; Liberzon et al., 2015).

### 2.4 Construction of weighted gene Co-expression network analysis (WGCNA) network for identifying key gene modules in NAFLD

To identify key co-expression gene modules associated with the pathogenesis of NAFLD, this study employed Weighted Gene Co-expression Network Analysis (WGCNA); following variance-based filtering of low-expression genes, transcriptomic data from both NAFLD patient and healthy control groups were integrated for co-expression network construction, whereby the optimal soft-thresholding power (β) was determined to ensure the network met scale-free topology criteria and the adjacency matrix was transformed into a Topological Overlap Matrix (TOM) to refine gene similarity metrics, followed by TOM-based dissimilarity hierarchical clustering (Ravasz et al., 2002); gene modules were initially identified using the dynamic tree cut algorithm and subsequently merged based on a module eigengene correlation threshold (≥0.25) to consolidate highly similar modules; the association strength between each module and NAFLD phenotypic traits was then evaluated by calculating Pearson correlation coefficients between module eigengenes and clinical traits, with the most significantly correlated modules selected for in-depth analysis; within the target module, hub genes were screened based on intramodular connectivity metrics, including module membership (MM) and gene significance (GS) relative to NAFLD-associated pathological traits.

### 2.5 Machine learning-based identification of IM-FT-related diagnostic biomarkers with signature gene validation and nomogram construction

Based on the expression profiles of IM-FT-related genes, the Least Absolute Shrinkage and Selection Operator (LASSO) regression model was first employed to screen key biomarkers significantly associated with disease status, where the optimal model structure was determined using the minimum λ value, with candidate genes exhibiting non-zero coefficients identified through LASSO coefficient plots and cross-validation curves (Maksimov et al., 2012); subsequently, expression data of key genes extracted from the training dataset were analyzed by Wilcoxon rank-sum test for differential expression between disease and control groups, visualized via violin plots; to validate screening reliability, differential expression was confirmed in validation datasets (GSE16470/GSE48452), followed by evaluation of each gene’s diagnostic performance using receiver operating characteristic (ROC) curves, with calculation of the area under the curve (AUC), 95% confidence interval, and optimal cutoff value (implemented via the pROC package) (Robin et al., 2011); ultimately, a nomogram prediction model incorporating key genes was constructed using the rms package, with predictive accuracy quantified through calibration curves.

### 2.6 Analysis of the immune microenvironment

To quantitatively assess the relative abundance of immune cells in NAFLD *versus* control samples, immune cell infiltration analysis was performed using the CIBERSORT algorithm based on predefined gene signature sets for 22 immune cell types, with individual sample immune composition profiles visualized via stacked bar plots; statistical significance of intergroup immune cell infiltration differences was evaluated through Wilcoxon rank-sum tests and presented in box plots; to investigate intrinsic associations between IM-FT-related biomarkers and immune infiltration, Spearman rank correlation coefficients were calculated, and statistically significant correlations after multiple test correction were screened and visualized using lollipop plots; concurrently, a heatmap of the Spearman correlation matrix among immune cells was constructed to elucidate potential synergistic or antagonistic interactions between distinct immune cell subsets, thereby systematically delineating regulatory networks linking immune microenvironment features with core metabolic pathways (Steen et al., 2020).

### 2.7 Identification of IM-FT molecular subtypes associated with NAFLD pathogenesis

Through unsupervised consensus clustering of the immune-metabolism functional transcriptome (IM-FT) expression profiles in NAFLD patients, this study aimed to systematically resolve their underlying molecular subtype structures; the clustering analysis was implemented using the “ConsensusClusterPlus” R package with a maximum cluster threshold set at 9 (maxK = 9), employing the Partitioning Around Medoids (PAM) algorithm and Euclidean distance as the similarity metric, while ensuring model stability and reproducibility through 50 iterative calculations; the optimal cluster number was determined by comprehensive evaluation of the cumulative distribution function (CDF) curve characteristics and quantitative assessment of the area under the curve variations (Wilkerson and Hayes, 2010); based on this molecular subtyping framework, the NAFLD cohort was stratified into IM-FT subtypes with significant heterogeneity (defined sequentially as Subtype 1, Subtype 2, etc.); to validate the biological rationality of the subtype classification, principal component analysis (PCA) was further performed to quantitatively evaluate the dispersion degree and spatial distribution patterns of subtype samples in the reduced-dimensional space, thereby confirming the presence of significant transcriptomic disparities among subtypes.

### 2.8 Molecular docking and molecular dynamics simulations between key NAFLD-associated genes and small molecules

To evaluate interactions between key NAFLD-associated target genes and potential therapeutic small molecules, this study employed an integrated computational strategy: starting with systematic literature mining to screen small-molecule ligands regulating core NAFLD targets, followed by acquisition of corresponding 3D protein structures from the Protein Data Bank (PDB); high-precision molecular docking simulations were then conducted using AutoDock4/AutoGrid4 to quantify ligand-receptor binding free energy (ΔG), with a docking energy ≤-5.0 kcal/mol set as the threshold for strong interactions; key molecular interaction patterns (hydrogen bonding/hydrophobic contacts) were resolved through PyMOL-based 3D visualization; to validate docking conformation stability and dynamic binding characteristics, molecular dynamics (MD) simulations using the AMBER19SB force field were further implemented: the bound complexes underwent 100-ns all-atom MD simulations in explicit TIP3P water molecules under physiological ionic concentration (0.15 M NaCl), with electrostatic interactions treated by the PME method while maintaining temperature/pressure at 310 K/1 bar, whereby system stability was evaluated via parameters including RMSD (overall conformational drift), RMSF (residue flexibility fluctuations), and radius of gyration (Rg); ultimately, trajectory-averaged binding free energy was calculated using the MM/PBSA method, enabling comprehensive screening of high-affinity conformationally stable candidates based on integrated docking and dynamics results to provide multi-scale molecular mechanistic evidence for NAFLD drug repurposing (Naqvi et al., 2018; Santos et al., 2019; Okimoto et al., 2009).

### 2.9 Single-cell RNA sequencing data acquisition and quality control

Based on the single-cell transcriptomic dataset (GSE225381), this study implemented comprehensive analysis using Seurat (v4.0) and Harmony (v0.1.0) packages; quality control procedures were executed with the following standards: filtration of aberrant cells exhibiting detected gene counts within [200, 5,000] or mitochondrial gene proportions >20% or ribosomal gene proportions >3%; data normalization employed the LogNormalize algorithm while concurrently filtering low-abundance genes (>2000 cells expressing) and removing doublets via DoubletFinder (v2.0.3); batch effect correction integrated the Harmony algorithm into principal component analysis (PCA), conducting dimensionality reduction based on the top 17 principal components; cellular clustering utilized Seurat’s FindNeighbors (distance metric: Euclidean) and FindClusters (resolution: 0.6) functions, with high-dimensional data visualization achieved through Uniform Manifold Approximation and Projection (UMAP) (Hoffman et al., 2018); cell type annotation leveraged the HumanPrimaryCellAtlas (v1.0) reference dataset with automated labeling through SingleR (v1.6.1); cluster-specific differentially expressed genes were defined using significance thresholds of log_2_-fold change >1 and Benjamini–Hochberg adjusted p-value <0.05, systematically resolving tissue cellular heterogeneity and immune microenvironment characteristics in NAFLD through multidimensional visualization techniques including complex heatmaps and dot plots (Xi et al., 2019).

### 2.10 Establishment of the NAFLD rat model

Four-week-old male Sprague-Dawley rats (n = 12) with specific pathogen-free (SPF) status and an initial body weight of (150 ± 10) g were randomly divided into two groups (n = 6 per group). All rats were supplied by Hunan Silaike Jingda Laboratory Animal Co., Ltd. (license number: SCXK (Xiang) 2024–00094). The standard chow diet and high-fat diet (HFD) were also procured from the same company (license number: SCXK (Xiang) 2020–0,006). The normal control group was fed a standard chow diet, while the model group was fed the HFD for a duration of 24 weeks to induce NAFLD, a well-established dietary intervention model for studying steatosis and metabolic dysfunction 25. All animals were housed in the Animal Facility of the Nanchang Research Institute of Sun Yat-sen University (license number: SYXK (Gan) 2023–0,014) under a controlled environment with regulated temperature, *ad libitum* access to food and water, and a standardized 12-h light/dark cycle. The animal experimental protocol was reviewed and approved by the Institutional Animal Care and Use Committee (IACUC) of the Nanchang Research Institute of Sun Yat-sen University (approval number: SYSUNC-IACUC-2023-0002). All procedures were conducted in strict accordance with the 3R (Replacement, Reduction, Refinement) principles for humane animal treatment.

### 2.11 Quantitative analysis of IM-FT-related biomarkers via qRT-PCR

Total RNA was extracted from liver tissues using a Tissue/Cell RNA Rapid Extraction Kit (Mei5 Biotechnology, China, Cat# MF036-01), followed by reverse transcription into cDNA using the “Chiliu” Fast RT Kit (Mei5 Biotechnology, Cat# MF949-01). Amplification of each sample was performed in a 20 μL reaction mixture employing the mRNA Fluorescence Quantitative PCR Detection Kit (Mei5 Biotechnology, Cat# MF013-01) on a real-time PCR system. The 2^−ΔΔCT^ method was applied to calculate fold-change values, with expression levels normalized to the endogenous control Gapdh (Glyceraldehyde-3-phosphate dehydrogenase), a well-established reference gene for metabolic studies in rodent models of NAFLD 9. All primer sequences used are listed in Table 1.

**TABLE 1:** The sequences of primers.

Genes	Forward primer (5′–3′)	Reverse primer (5′–3′)
ERN1	AAT​GGC​GAT​GGA​CTG​GTG​GTA	TAC​AGA​GTG​GGC​GTC​AGT​TTG​C
SLC11A1	CAG​GCA​AGG​ATC​TGG​GTG​AAA​T	AGG​AGA​TAG​CCG​TTC​CGA​TGA​C
GAPDH	CTG​GAG​AAA​CCT​GCC​AAG​TAT​G	GGT​GGA​AGA​ATG​GGA​GTT​GCT

### 2.12 Statistical analysis and visualization

Data analysis and visualization were performed using R software (version 4.3.3) and GraphPad Prism 9. Differences between two groups were assessed using paired t-tests for normally distributed data and Wilcoxon rank-sum tests for non-normally distributed data, with statistical significance defined as p < 0.05.

## 3 Results

### 3.1 Identification and analysis of NAFLD-Associated differentially expressed genes

**FIGURE 2:**
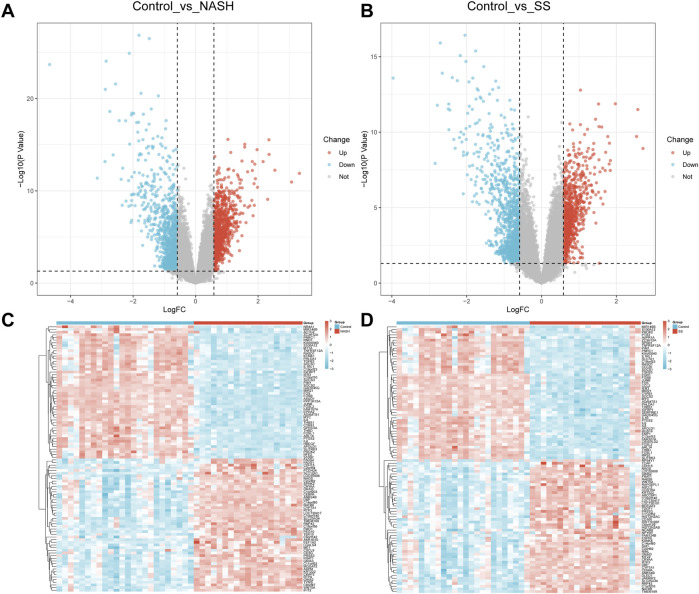
Identification and Visualization of NAFLD-Associated Differentially Expressed Genes. **(A,B)** Volcano plots displaying differentially expressed genes (DEGs) between NASH/steatotic (SS) groups *versus* control group (red: upregulated genes; blue: downregulated genes; gray: non-significant genes); **(C,D)** Heatmaps illustrating expression patterns of the most significant DEGs in NASH/SS groups compared to controls.

This study analyzed NAFLD-related transcriptomic datasets from the Gene Expression Omnibus (GEO) database, encompassing 24 normal control samples, 20 steatotic (SS) samples, and 19 non-alcoholic steatohepatitis (NASH) samples. The data processing followed the framework established in murine NAFLD transcriptome studies to ensure methodological consistency. Differential expression analysis was conducted on normalized data using the limma package, with cutoff criteria set as |log_2_ fold change (FC)| > 0.585 and p < 0.05—consistent with the effect size thresholds employed in human NAFLD cohort analyses. Comparisons between the control and NASH groups identified 2,131 differentially expressed genes (DEGs), including 1,158 upregulated and 973 downregulated genes. In contrast, 2,074 DEGs (1,191 upregulated; 883 downregulated) were detected in the control vs. SS comparison. These DEGs primarily reflected transcriptional perturbations in lipid metabolism and inflammatory pathways, aligning with observations reported in genetic and dietary NAFLD models. Volcano plots (Figures 2 A,B) visualized these expression patterns, with red dots representing upregulated genes, blue dots indicating downregulated genes, and gray dots denoting non-significant genes. Heatmaps (Figures 2 C,D) further delineated the expression profiles of the most significantly dysregulated DEGs in SS/NASH groups relative to controls. These visualizations revealed distinct intergroup segregation patterns, which strongly validated the reliability of the differential expression results—consistent with the sample stratification observed in human liver transcriptomic studies focusing on NAFLD progression.

### 3.2 Identification and functional enrichment analysis of key IM-FT-related differentially expressed genes

**FIGURE 3:**
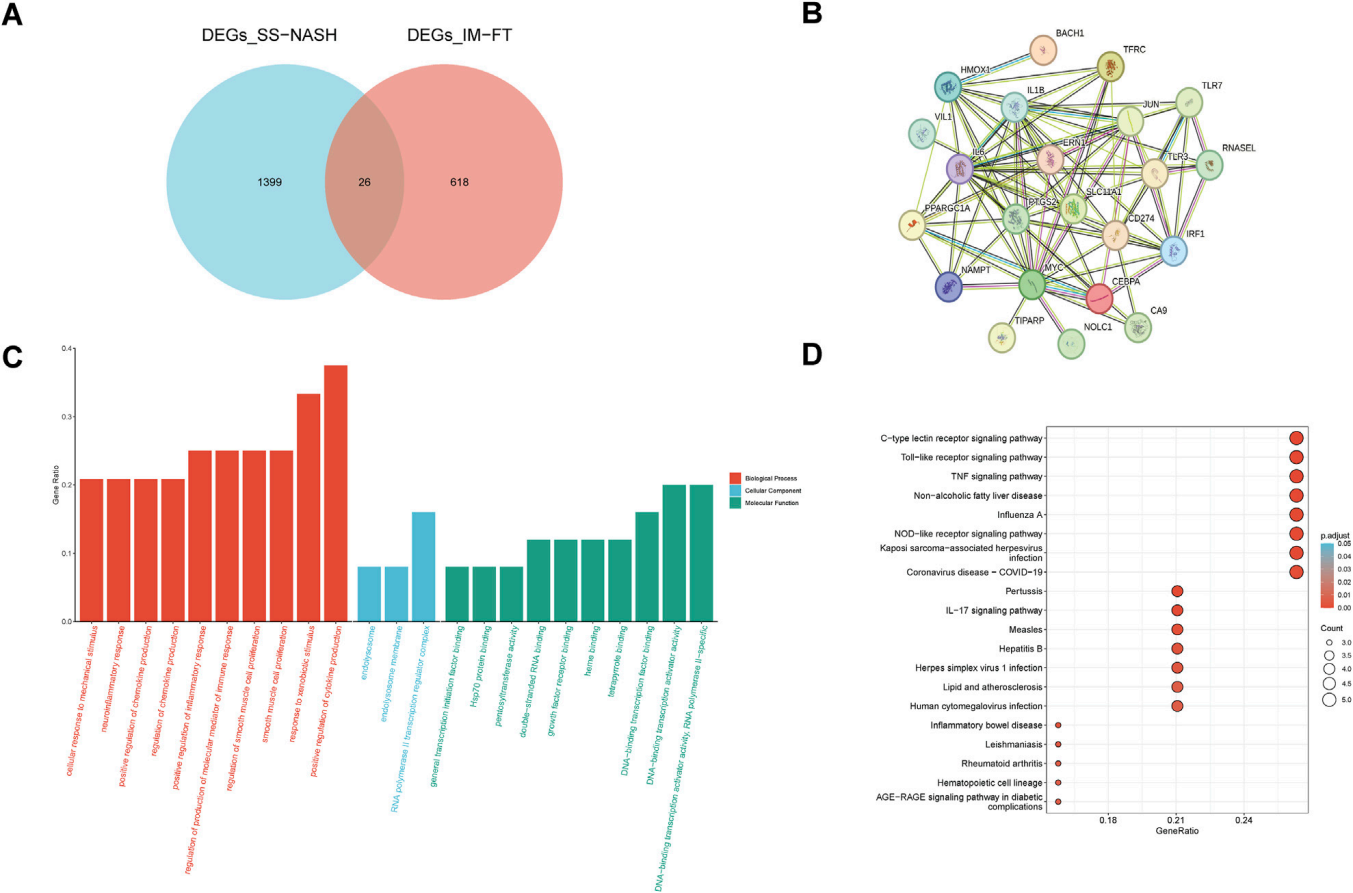
Identification and Functional Analysis of Differentially Expressed IM-FT in NAFLD. **(A)** Venn diagram showing intersection between SS/NASH-associated DEGs (blue) and IM-FT gene sets (red), identifying 26 key DE_IM-FTs; **(B)** Protein-protein interaction network of the 26 key DE_IM-FTs; **(C)** GO enrichment analysis displaying significant terms for biological processes (BP), cellular components (CC), and molecular functions (MF); **(D)** KEGG pathway enrichment analysis of signaling pathways enriched by DE_IM-FTs.

To further explore the association between differentially expressed genes common to both SS and NASH samples (DEGs_SS-NASH) and those linked to iron metabolism and ferroptosis (DEGs_IM-FT), we performed intersection analyses. First, overlapping genes between DEGs_SS-NASH and DEGs_IM-FT were identified, yielding 644 shared differentially expressed genes (Supplementary Figure S1A). Additionally, intersection of DEGs from limma analyses of SS (DEGs_limma-SS) and NASH (DEGs_limma-NASH) groups revealed 1,425 overlapping genes (Supplementary Figure S1B). Further, a refined intersection between DEGs_SS-NASH and DEGs_IM-FT identified 26 core overlapping genes (designated DE_IM-FTs; Figure 3A).

Subsequently, a protein-protein interaction (PPI) network was constructed for these 26 DE_IM-FTs, revealing intensive interconnections among them (Figure 3B). To elucidate their functional roles, Gene Ontology (GO) and Kyoto Encyclopedia of Genes and Genomes (KEGG) enrichment analyses were performed. GO enrichment results (Figure 3C) showed that these 26 DE_IM-FTs were significantly enriched in biological processes such as cellular response to mechanical stimulus, neuroinflammatory response, and heme binding. Accumulating evidence indicates that inflammatory and immune responses can elevate reactive oxygen species (ROS) levels and promote iron overload, while transcription factors regulate antioxidant defenses; notably, core iron metabolism-related processes (e.g., heme binding) directly supply iron sources, thereby triggering ferroptosis—supporting the involvement of these DE_IM-FTs in iron metabolism/ferroptosis pathways (Chen et al., 2022). KEGG pathway enrichment analysis (Figure 3D) further demonstrated that DE_IM-FTs may regulate pathways closely linked to NAFLD pathogenesis, including immune-inflammatory responses, infectious diseases, and metabolic disorders. Iron, known to act as a catalyst for inflammation and a trigger for cell death, accumulates in such pathological contexts to induce lipid peroxidation, ultimately driving ferroptosis. Collectively, these findings suggest that the identified key DE_IM-FTs provide novel insights into the molecular mechanisms underlying the crosstalk between iron metabolism/ferroptosis and NAFLD, offering promising directions for further investigation.

### 3.3 Weighted gene Co-Expression network analysis (WGCNA) of NAFLD-Associated gene modules

**FIGURE 4:**
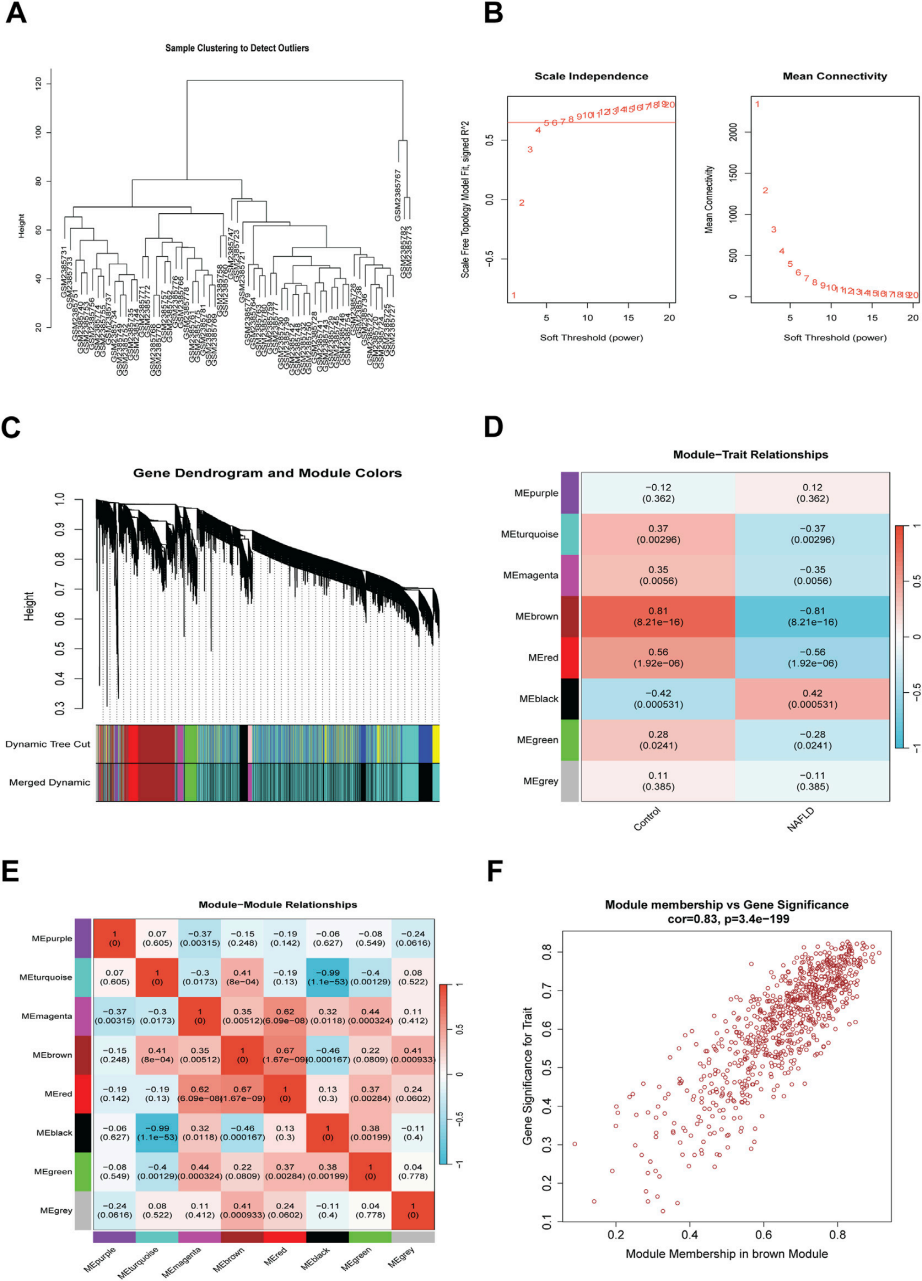
Identification of Key Gene Modules Associated with NAFLD by WGCNA. **(A)** Sample clustering analysis for outlier detection; **(B)** Analysis of scale-free topology fit index for soft threshold determination; **(C)** Gene dendrogram with identified modules following dynamic tree cutting; **(D)** Module-trait correlation analysis displaying relationships between modules and clinical traits; **(E)** Module-module correlation analysis; **(F)** Scatter plot of module membership *versus* gene significance in the brown module.

To further explore the potential molecular mechanisms driving NAFLD pathogenesis, weighted gene co-expression network analysis (WGCNA) was utilized to identify key disease-associated gene modules. Initially, cluster analysis of 63 samples was performed, with no significant outliers identified (Figure 4A). Subsequently, the scale-free topology fit index (*R* ^2^) and mean connectivity were calculated across a range of soft-thresholding powers, and a soft threshold power of β = 15 was selected for co-expression network construction based on these metrics (Figure 4B). Genes were clustered using the dynamic tree cut algorithm, with highly similar modules merged to refine the results (Figure 4C), ultimately yielding eight distinct co-expression modules. Correlation analyses were then performed to assess associations between each module and disease status. As shown in Figure 4D, the brown module exhibited a strong positive correlation with the control group (r = 0.81, p = 8.21e-16) and a negative correlation with the NAFLD group (r = −0.51, p = 8.21e-16); the red module was positively correlated with controls (r = 0.56, p = 1.92e-06) and negatively correlated with NAFLD (r = −0.56, p = 1.92e-06); the black module showed a negative correlation with controls (r = −0.42, p = 0.000531) and a positive correlation with NAFLD (r = 0.42, p = 0.000531); the turquoise module was positively correlated with controls (r = 0.37, p = 0.00296) and negatively correlated with NAFLD (r = −0.37, p = 0.00296); and the magenta module exhibited a positive correlation with controls (r = 0.35, p = 0.0056) and a negative correlation with NAFLD (r = −0.35, p = 0.0056). These findings suggest that these five modules may play critical roles in NAFLD progression.

Further, module correlation analysis revealed high independence among modules, with no statistically significant inter-module correlations detected (Figure 4E). To identify disease-related hub genes, a scatter plot of module membership *versus* gene significance was generated for the brown module (Figure 4F), which showed a significant positive correlation (r = 0.83, p = 3.4e-199). This strong correlation indicates that the collective effects of genes within the brown module strongly contribute to their association with NAFLD. Consequently, genes from the brown, red, and black modules were selected as candidate key genes for subsequent bioinformatic analyses.

### 3.4 Screening and identification of characteristic IM-FT biomarkers for NAFLD

**FIGURE 5:**
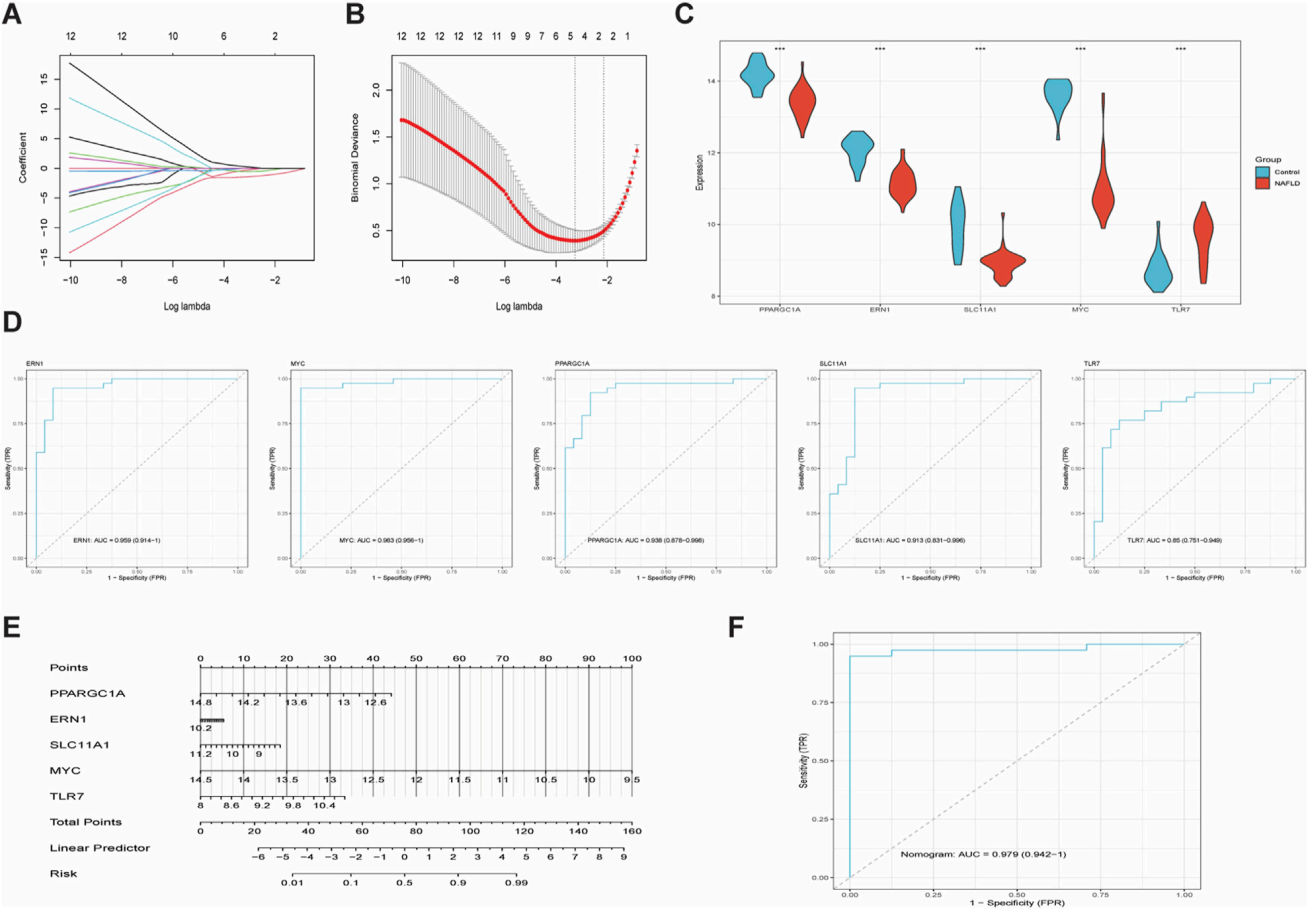
Machine Learning Algorithms and Diagnostic Validation for Identifying Characteristic IM-FT Biomarkers in NAFLD. **(A,B)** Variable selection based on LASSO algorithm showing coefficient profile and partial likelihood deviance; **(C)** Expression profiles of ERN1, MYC, PPARGC1A, SLC11A1, and TLR7 in control *versus* NAFLD groups; **(D)** ROC curves of five candidate biomarkers; **(E)** Nomogram model constructed from the five-gene signature; **(F)** ROC curve of the nomogram prediction model.

To identify overlapping genes between key modules derived from WGCNA and DE_IM-FT, Venn diagram analysis was performed, yielding 15 shared genes (Supplementary Figure S1C). To further screen characteristic iron metabolism/ferroptosis (IM-FT)-related biomarkers associated with NAFLD, feature selection was conducted using the LASSO algorithm (Figures 5A,B). Following determination of the optimal log(λ) value, five feature variables—ERN1, MYC, PPARGC1A, SLC11A1, and TLR7—were identified based on L1 regularization. The diagnostic efficacy of these five IM-FT-related biomarkers for NAFLD was subsequently evaluated. In the training set, compared with the control group, the NAFLD group showed significantly downregulated expression of ERN1, MYC, PPARGC1A, and SLC11A1, whereas TLR7 expression was significantly upregulated (Figure 5C). Receiver operating characteristic (ROC) curve analysis revealed AUC values of 0.959, 0.983, 0.938, 0.913, and 0.85 for ERN1, MYC, PPARGC1A, SLC11A1, and TLR7, respectively (Figure 5D), supporting their robust diagnostic utility for NAFLD. A nomogram model constructed using these five genes yielded an AUC of 0.979 (Figures 5 E,F), further validating its potential for clinical application in NAFLD diagnosis. In the validation set, consistent with the training set, ERN1 and SLC11A1 expression remained significantly lower in the NAFLD group compared with controls, whereas no significant differences were observed for PPARGC1A, MYC, or TLR7 (Supplementary Figure S1D). ROC analysis in the validation set showed AUC values of 0.855, 0.667, 0.772, 0.89, and 0.681 for ERN1, MYC, PPARGC1A, SLC11A1, and TLR7, respectively (Supplementary Figure S1E). Although these values were slightly lower than those in the training set, diagnostic value was still evident—particularly for ERN1 and SLC11A1. In summary, this study identifies ERN1 and SLC11A1 as characteristic IM-FT-related biomarkers, which are significantly differentially expressed in NAFLD and exhibit strong diagnostic performance. Notably, their integration into the five-gene nomogram model further enhances diagnostic efficacy.

### 3.5 Immune cell infiltration analysis and the Correlation of Characteristic NAFLD IM-FT with immune microenvironment features

**FIGURE 6:**
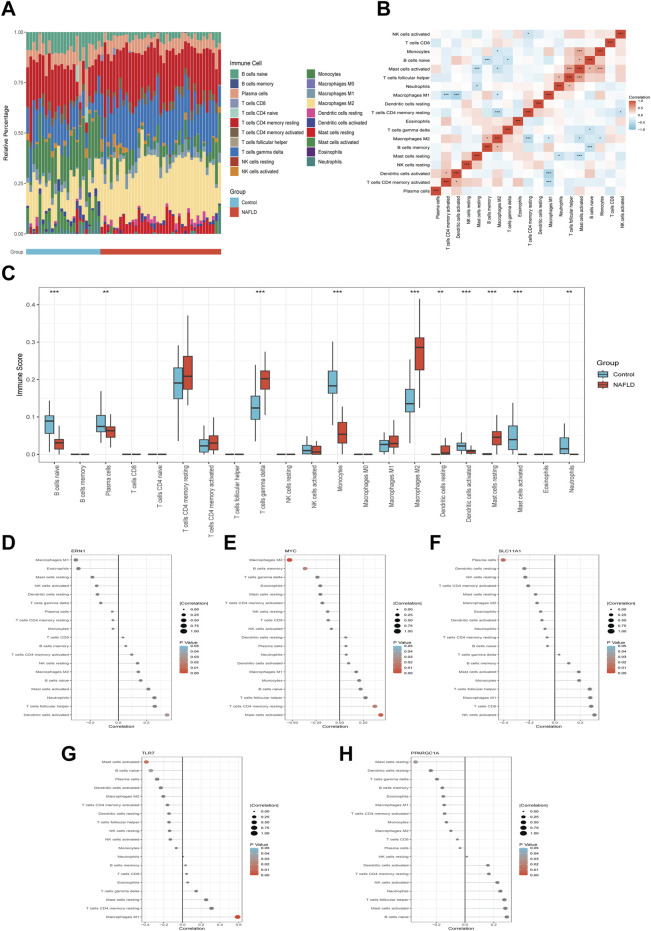
Immune Infiltration Analysis in NAFLD and Correlation of Characteristic IM-FT. **(A)** CIBERSORT analysis of 22 immune cell types in the control and NAFLD groups. **(B)** Correlation heatmap among immune cell subtypes. **(C)** Differential immune cell infiltration between the control (blue) and NAFLD (red) groups. **(D–H)** Correlation analysis of ERN1 **(D)**, MYC **(E)**, SLC11A1 **(F)**, TLR7 **(G)**, and PPARGC1A **(H)** with immune cell types. Dot size represents the magnitude of the correlation coefficient; color indicates statistical significance (*P < 0.05; **P < 0.01; ***P < 0.001).

To characterize the immune cell infiltration landscape of NAFLD, we utilized the CIBERSORT algorithm to quantify the compositional profiles of 22 immune cell subsets in control and NAFLD groups. Cumulative bar graphs visualized the relative proportions of each immune cell subtype (Figure 6A), revealing marked compositional alterations in NAFLD—specifically, significant changes in the proportions of activated CD4 memory T cells, plasma cells, gamma delta T cells, and M2 macrophages. Heatmap analysis further uncovered robust correlations among immune cell subtypes. For instance, activated CD4 memory T cells exhibited a strong negative correlation with M1 macrophages, while resting macrophages showed a prominent positive correlation with resting mast cells (Figure 6B). Differential comparison confirmed that the NAFLD group had a marked and significant increase in the proportions of gamma delta T cells, M2 macrophages, and resting mast cells (p < 0.001), accompanied by a significant reduction in plasma cells, resting macrophages, and naive B cells (p < 0.01). These observations offer critical insights into the distinct immune microenvironment features of NAFLD (Figure 6C). To elucidate the crosstalk between IM-FT-related biomarkers (ERN1, MYC, SLC11A1, TLR7, and PPARGC1A) and the immune microenvironment, Pearson correlation analysis was performed (Figures 6D–H). ERN1 exhibited a positive correlation with activated dendritic cells. MYC was positively associated with activated mast cells and resting CD4 memory T cells, while showing a negative correlation with M2 macrophages and memory B cells. SLC11A1 displayed a negative correlation with plasma cells. TLR7 demonstrated positive correlations with cell subsets such as M1 macrophages and negative correlations with others (e.g., activated mast cells). In contrast, PPARGC1A showed a negative correlation with resting mast cells. Collectively, these results imply that IM-FT-related pathways may modulate NAFLD development and progression by regulating the abundance and functional status of specific immune cell subsets within the hepatic immune microenvironment.

### 3.6 Identification of characteristic IM-FT-related molecular signatures and functional characterization of two subtypes in NAFLD

**FIGURE 7:**
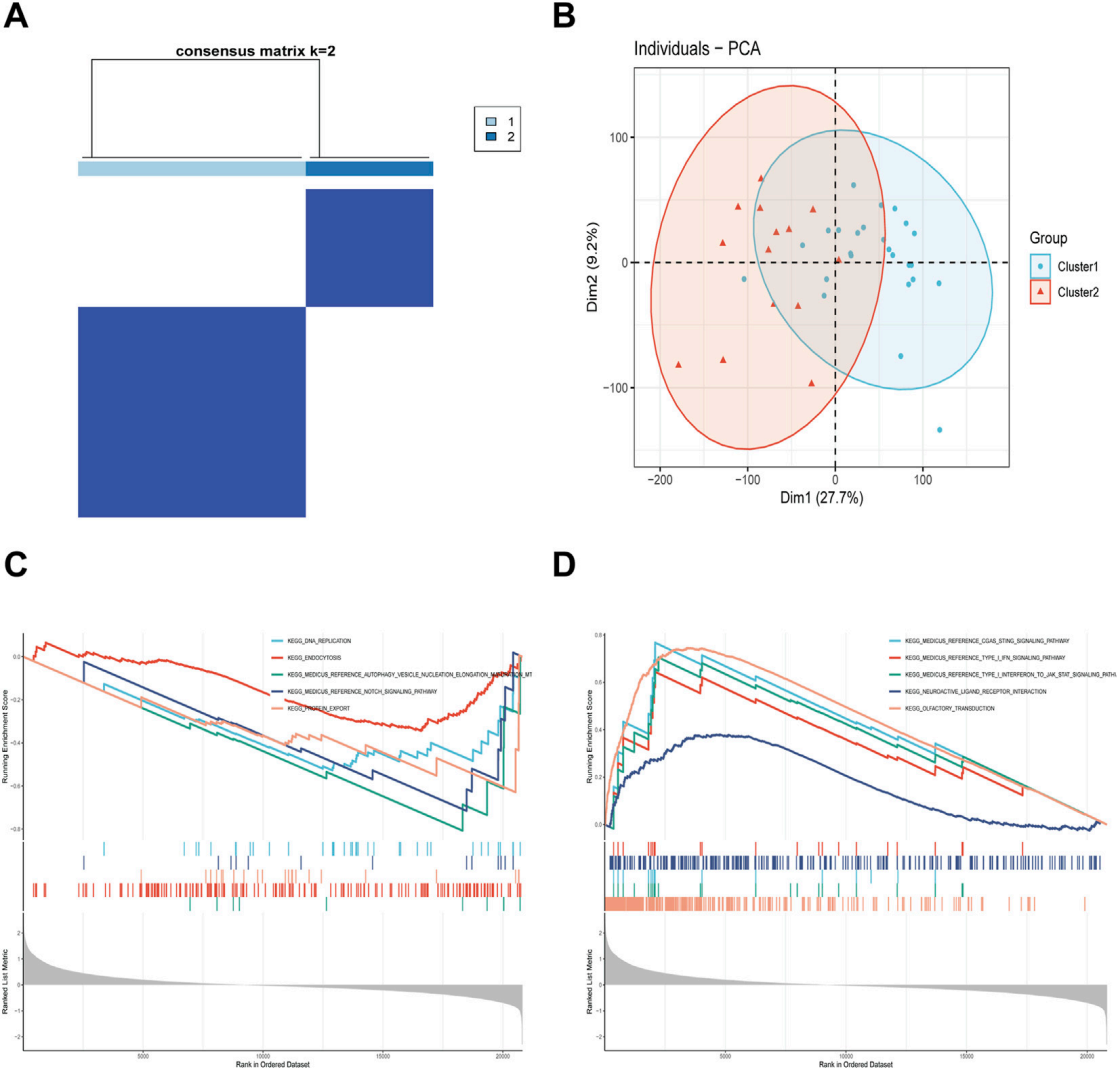
Analysis of Characteristic IM-FT-Related Molecular Patterns in NAFLD. **(A)** Consensus clustering matrix at k = 2. **(B)** PCA scatter plot illustrating sample distribution. **(C,D)** GSEA pathways enriched in Subtype 1 **(C)** and Subtype 2 **(D)**.

To elucidate the molecular patterns of characteristic IM-FT in NAFLD, consensus clustering analysis was performed on the samples. Based on the consensus matrix (Figure 7A), the optimal cluster number was determined to be k = 2. PCA further demonstrated clear separation between the two subtypes, designated as Subtype 1 and Subtype 2 (Figure 7B). GSEA further highlighted the biological disparities between the two subtypes. In Subtype 1, pathways such as DNA replication, autophagosome assembly, and NOTCH signaling were significantly enriched (Figure 7C). Conversely, Subtype 2 exhibited enrichment in pathways associated with neuroactive ligand-receptor interactions and cGAS-STING signaling pathway (Figure 7D). These results reveal significant functional differences between the two subtypes, providing insights into the distinct molecular and biological mechanisms underlying NAFLD.

### 3.7 Molecular docking and molecular dynamics simulation analysis of characteristic IM-FT targeted proteins

**FIGURE 8:**
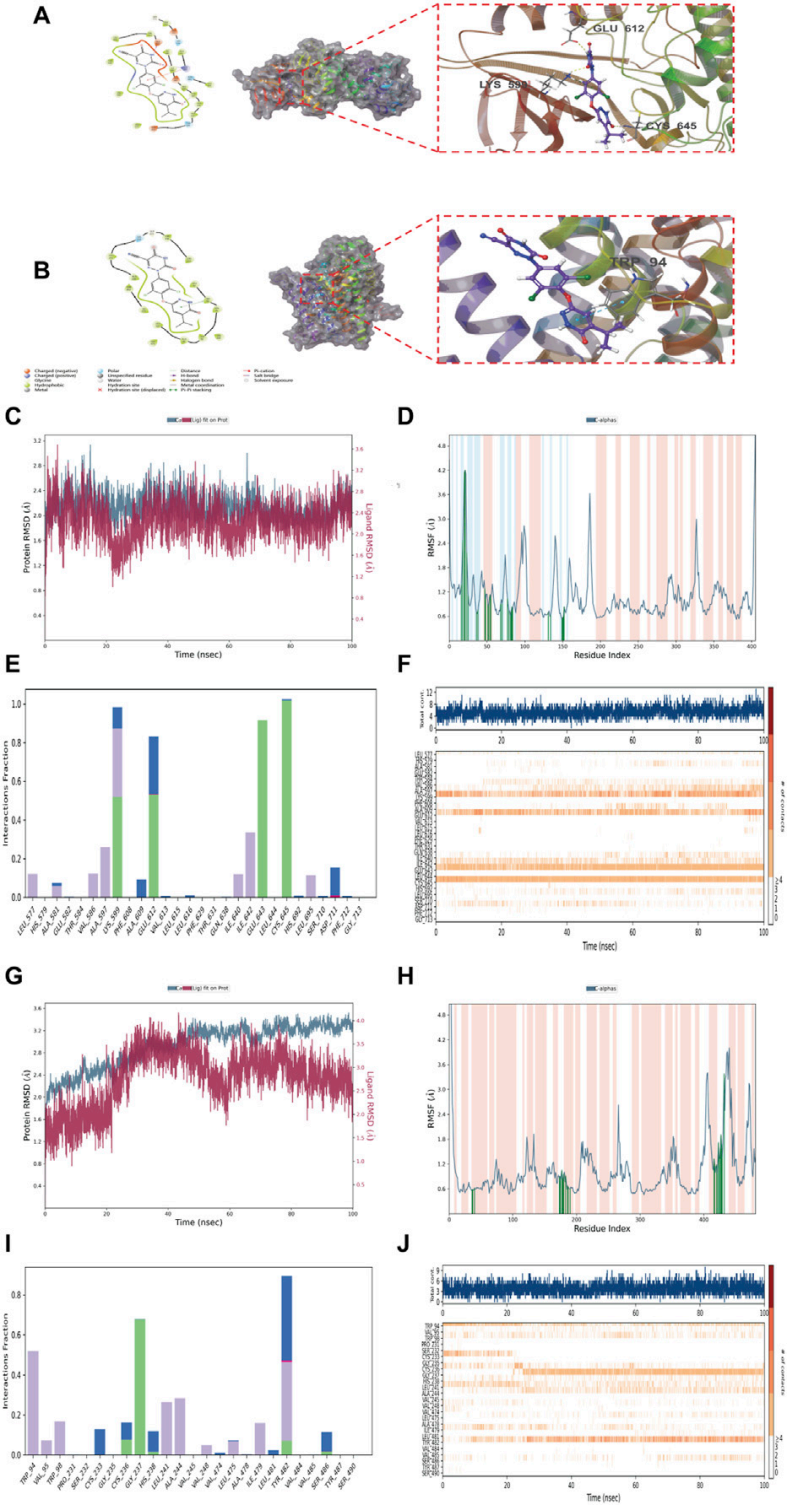
Molecular Docking and Molecular Dynamics Simulation Analysis of Characteristic IM-FT Targeted Proteins. **(A,B)** Binding interactions between Resmetirom and ERN1 **(A)** and SLC11A1 **(B)**; **(C–F)** Molecular dynamics simulation analysis of Resmetirom with ERN1 protein: RMSD analysis **(C)**, RMSF analysis **(D)**, hydrogen bond analysis **(E)**, interaction analysis **(F)**; **(G–J)** Molecular dynamics simulation analysis of Resmetirom with SLC11A1 protein: RMSD analysis **(G)**, RMSF analysis **(H)**, hydrogen bond analysis **(I)**, interaction analysis **(J)**.

To identify potential small-molecule therapeutics for NAFLD, recent clinical trials have shown that Resmetirom effectively reduces hepatic lipid content in NAFLD patients by selectively activating hepatic thyroid hormone receptor β (THR-β), promoting lipid metabolism, decreasing hepatic fat accumulation, and improving insulin sensitivity, while further facilitating the resolution of non-alcoholic steatohepatitis (NASH) without exacerbating fibrosis (Dutta et al., 2024; Vidal-Cevallos et al., 2023). In preclinical animal models, Resmetirom administration significantly decreased liver weight, hepatic lipid content, plasma alanine aminotransferase (ALT) activity, and hepatic/plasma cholesterol concentrations, consequently improving NAFLD activity scores (NAS) (Kannt et al., 2021), which suggests that Resmetirom may mitigate lipid peroxidation and its anti-inflammatory properties (e.g., suppression of tumor necrosis factor-α [TNF-α] and interleukin-1β [IL-1β]) may attenuate inflammation-driven dysregulation of iron metabolism. THR-β activation enhances mitochondrial β-oxidation, and given that mitochondria play a central role in ferroptosis execution, these pathways may crosstalk through energy metabolic cascades (Caddeo et al., 2021). Molecular docking and molecular dynamics (MD) simulations were conducted to evaluate Resmetirom as a potential therapeutic candidate, and comprehensive analysis of XP docking and molecular mechanics generalized Born surface area (MM-GBSA) results showed that Resmetirom binds to ERN1 and SLC11A1 with docking scores of −7.773 and −6.644, respectively, and MM-GBSA binding energies of −49.04 and −52.77 kcal/mol, with low docking scores and binding energies reflecting stable interactions between Resmetirom and these two proteins (Figures 8A,B); XP Gscores <−6 are indicative of stable ligand-protein binding, whereas MM-GBSA dG Bind values <−30 kcal/mol denote favorable binding stability, and both criteria were satisfied, confirming that Resmetirom exhibits favorable binding affinity for iron metabolism-ferroptosis (IM-FT) target proteins. A 100 ns MD simulation of Resmetirom-ERN1 complexes was performed to assess conformational stability using root mean square deviation (RMSD; Figure 8C), which revealed minor fluctuations after 35 ns, indicating system equilibrium, while root mean square fluctuation (RMSF) analysis characterized local protein flexibility with peaks representing high mobility in residues 15–25, 85–105, 135–150, and 180–190 (Figure 8D); key interaction residues (LYS599, GLU612, GLU643, CYS645) sustained hydrogen bonds, hydrophobic interactions, water bridges, and ionic bonds throughout the simulation (Figure 8E), and interaction timeline analysis (Figure 8F) verified persistent interactions (deep orange) with these residues. For Resmetirom-SLC11A1 complexes, RMSD stability was attained after 80 ns (Figure 8G), and RMSF analysis showed high flexibility in residues 265–270, 390–420, and 430–450 (Figure 8H), with key interacting residues (TRP94, GLY237, TYR482) primarily forming hydrogen bonds, hydrophobic interactions, and water bridges (Figure 8I) and the sustained nature of these interactions confirmed by contact timeline analysis (Figure 8J). Collectively, these findings suggest that Resmetirom has the potential to reverse IM-FT-related expression perturbations, thereby inhibiting or arresting NAFLD progression.

### 3.8 Single-cell transcriptomic analysis of NAFLD

**FIGURE 9:**
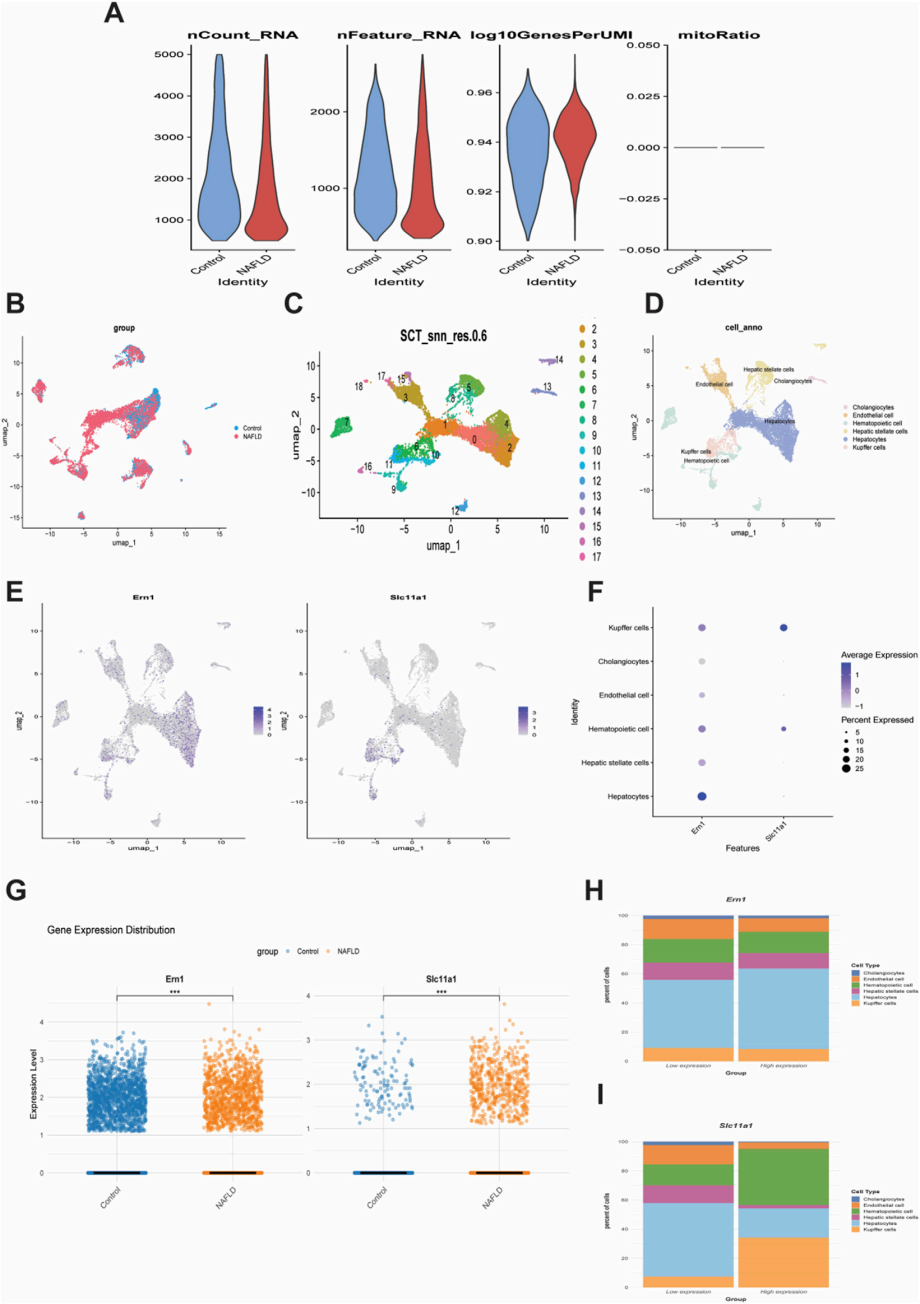
Single-Cell Transcriptomic Analysis of NAFLD. **(A)** Sample quality control plot. **(B)** UMAP plot displaying cell distribution in control and NAFLD groups. **(C)** Cell clustering based on transcriptomic profiles. **(D)** Identification of six major cell types. **(E)** Cellular distribution of ERN1 and SLC11A1 specific marker genes. **(F)** Dot plot showing ERN1 and SLC11A1 expression across cell types. **(G)** Comparison of ERN1 and SLC11A1 expression between NAFLD and control groups (***P < 0.001). **(H–I)** Cell type composition based on ERN1 and SLC11A1 expression.

Single-cell RNA sequencing (scRNA-seq) analysis was performed on NAFLD samples. Initial quality control of the samples (Figure 9A) followed by dimensionality reduction via UMAP visualized distinct cellular distribution patterns between the control and NAFLD groups (Figure 9B). Subsequent analysis identified 17 distinct subclusters (Figure 9C) categorized into six major cell types based on signature gene expression: cholangiocytes, endothelial cells, hematopoietic cells, hepatic stellate cells, hepatocytes, and Kupffer cells (Figure 9D). Cellular distributions of ERN1 and SLC11A1 marker genes were mapped (Figure 9E), while expression dot plots revealed differential expression patterns of ERN1 and SLC11A1 across these cell types (Figure 9F)—notably, ERN1 showed high expression in hepatocytes and Kupffer cells, whereas SLC11A1 was primarily expressed in Kupffer cells. Comparative analysis demonstrated significantly higher expression of both ERN1 and SLC11A1 in NAFLD *versus* controls (P < 0.001, Figure 9G). Stratification of cells based on ERN1 and SLC11A1expression levels (high vs. low) further revealed substantial differences in cell type composition (Figures 9H,I). These findings indicate that ERN1 and SLC11A1 may contribute to NAFLD pathogenesis and progression by modulating responses within specific cellular populations.

### 3.9 qRT-PCR validation in NAFLD rat liver tissues

**FIGURE 10:**
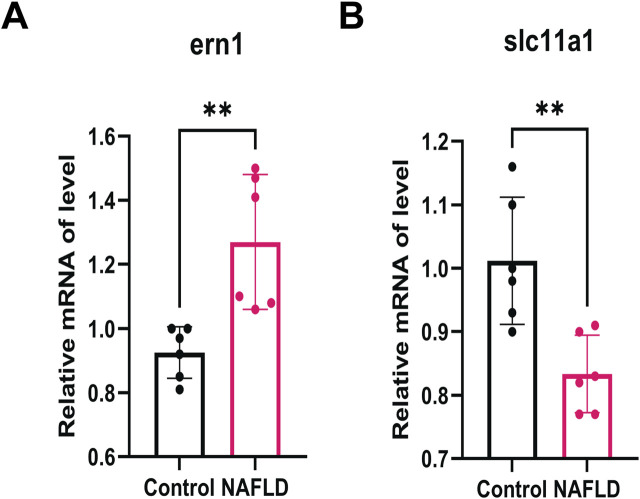
qRT-PCR Validation of Characteristic IM-FT Target Proteins Validation of ERN1 **(A)** and SLC11A1 **(B)** expression via qRT-PCR in NAFLD *versus* control groups.

Compared with the control group, we found a significant downregulation of the two hub genes (ERN1 and SLC11A1) in the NAFLD group, suggesting that their reduced expression may serve as potential risk factors for NAFLD. To validate the expression patterns of iron metabolism-ferroptosis (IM-FT)-related genes in NAFLD, a rat NAFLD model was established via high-fat diet (HFD) feeding, and quantitative real-time polymerase chain reaction (qRT-PCR) was conducted to assess gene expression in NAFLD and normal rat liver tissues. The results showed that *ern1* expression was significantly upregulated in the NAFLD group compared with the control group (P < 0.01, Figure 10A), whereas *slc11a1* expression was markedly decreased in the NAFLD group (Figure 10B). The expression pattern of SLC11A1 in the experimental model was consistent with the trends observed in our prior bioinformatics analysis and external validation datasets, thereby providing robust validation and further supporting the promising potential of SLC11A1 as a biomarker for NAFLD.

## 4 Discussion

NAFLD stands as one of the most prevalent chronic liver disorders globally, with its incidence escalating steadily year by year. Pathologically, NAFLD is characterized by excessive hepatic lipid accumulation (steatosis), hepatocellular injury, and an elevated susceptibility to progression toward liver fibrosis and hepatocellular carcinoma (HCC). This disease not only poses a severe threat to individual health but also inflicts a heavy economic burden on healthcare systems worldwide. While current management strategies—including lifestyle modifications and pharmacotherapies—are available, there remains a paucity of targeted therapeutic agents and robust efficacy assessment criteria for NAFLD (Britton et al., 2016; Sui et al., 2024). Therefore, in-depth investigations into the pathogenic mechanisms and identification of potential biomarkers for NAFLD carry profound clinical implications. The present study aimed to identify differentially expressed genes (DEGs) associated with iron metabolism and ferroptosis in NAFLD, thereby providing novel insights into the underlying disease mechanisms. By capitalizing on data retrieved from the Gene Expression Omnibus (GEO) database and integrating bioinformatics methodologies, our research circumvented the inherent limitations of traditional experimental studies, enabling a more comprehensive characterization of the molecular landscape of NAFLD. Our findings revealed that ERN1 and SLC11A1 serve as key regulatory molecules in NAFLD, with significant associations with characteristic features of the hepatic immune microenvironment. A notable innovation of this study lies in the adoption of multi-method cross-validation to confirm the reliability of candidate biomarkers. Furthermore, we integrated molecular docking and molecular dynamics simulations to identify Resmetirom, a small-molecule compound that may exert therapeutic potential against NAFLD by targeting the iron metabolism-ferroptosis (IM-FT) axis. Notably, the establishment of a NAFLD rat model, followed by quantitative real-time polymerase chain reaction (qRT-PCR) analysis, validated the differential expression patterns of these key molecular biomarkers at the tissue level. This provided experimental corroboration that reinforces the robustness of our findings, and underscores the considerable potential of SLC11A1 as both a diagnostic biomarker and a therapeutic target for NAFLD.

ERN1 (Endoplasmic Reticulum to Nucleus Signaling 1), located on human chromosome 8, encodes a key endoplasmic reticulum stress sensor. Structurally, it features an enzymatic domain possessing both endonuclease and kinase activities (Minchenko et al., 2024a). It plays a critical role in regulating gene expression and cellular stress responses, with its relevance in the tumor microenvironment increasingly recognized; its significance in glioblastoma is now emerging (Minchenko et al., 2024b). ERN1 contributes substantially to the pathophysiology of NAFLD. As an endoplasmic reticulum stress sensor, dysfunctional ERN1 can lead to increased apoptosis and hepatic lipid accumulation. Studies indicate that ERN1 activation is associated with hepatocyte steatosis and inflammatory responses, suggesting its potential as a therapeutic target in NAFLD progression (Lebeaupin et al., 2018). SLC11A1 (Solute Carrier Family 11 Member 1), situated on chromosome 2, is another important transporter primarily expressed in monocytes. This transmembrane protein functions in metal ion transport, particularly playing a pivotal role in restricting pathogen growth (Wu et al., 2024b). By modulating intracellular iron levels, it influences hepatic iron homeostasis, thereby affecting liver inflammation and oxidative damage—providing a mechanistic basis for its potential as a prognostic biomarker in NAFLD (Banerjee et al., 2025). ERN1 activation may synergistically contribute to NAFLD progression by regulating SLC11A1 expression, subsequently modulating iron metabolism and lipid deposition, indicating a potential interplay between these pathways in disease pathogenesis.

Resmetirom, a liver-targeted THR-β agonist, has been demonstrated to effectively reduce hepatic fat accumulation, ameliorate liver dysfunction, and improve liver fibrosis [12], while also exhibiting favorable safety and tolerability in clinical trials (Keam, 2024; Guirguis et al., 2025). Its mechanism of action primarily involves the activation of THR-β, leading to the upregulation of mitochondrial enzymes such as carnitine palmitoyltransferase 1 (CPT1), promotion of fatty acid β-oxidation, improved hepatic metabolism, and reduction of liver inflammation and fibrosis (Kannt et al., 2021; Vidal-Cevallos and Chávez-Tapia, 2024), thereby effectively decreasing intrahepatic lipid deposition (Bejitual et al., 2024). Given that mitochondria are central to the execution of ferroptosis, these mechanisms may interact through energy metabolism pathways (Gan, 2021). Molecular docking and dynamics simulations revealed that Resmetirom exhibited XP docking scores of −7.773 and −6.644 with ERN1 and SLC11A1, respectively, and MM-GBSA binding free energies of −49.04 kcal/mol and −52.77 kcal/mol. The docking scores (XP Gscore) were both below −6, and the binding free energies (MM-GBSA dG Bind) were substantially lower than −30 kcal/mol, indicating stable binding with both target proteins. A 100 ns MD simulation of ERN1 showed that the RMSD reached equilibrium after 35 ns; RMSF analysis indicated high flexibility in residue regions 15–25AA, 85–105AA, 135–150AA, and 180–190AA; key interacting residues—LYS599, GLU612, GLU643, and CYS645—maintained binding stability via hydrogen bonding, hydrophobic interactions, and water bridges. Simulation of SLC11A1 demonstrated RMSD stability after 80 ns, with significant fluctuations in regions 265–270AA, 390–420AA, and 430–450AA; essential residues TRP94, GLY237, and TYR482 facilitated binding through hydrogen bonds, hydrophobic forces, and water bridges. In summary, the stable binding of Resmetirom with ERN1 and SLC11A1 suggests its potential to mitigate NAFLD progression by modulating IM-FT target protein expression. Single-cell transcriptomic analysis further elucidated cell-type-specific expression patterns of these genes within the NAFLD microenvironment. Single-cell RNA sequencing (scRNA-seq) revealed significant heterogeneous expression of these genes across different cellular populations in NAFLD, highlighting functional diversity among cells and providing a novel cellular perspective on disease progression. Studies indicate that specific cell types, such as hepatocytes and macrophages, play crucial roles in the immune microenvironment of NAFLD, particularly in regulating local inflammatory responses and cell death. Moreover, the distinct contributions of various cell types to NAFLD pathogenesis warrant further investigation, potentially informing future therapeutic strategies. Quantitative reverse transcription polymerase chain reaction (qRT-PCR) validated expression differences of key protein biomarkers at the tissue level, providing experimental evidence that enhances the credibility of the findings and more robustly supports the greater potential of SLC11A1 as a biomarker for NAFLD. Future research should prioritize the translation of these experimental results into clinical strategies to improve therapeutic outcomes for NAFLD patients.

This study is not without limitations. First and foremost, while bioinformatics approaches enabled the identification of differentially expressed genes (DEGs) associated with NAFLD, experimental validation via quantitative real-time polymerase chain reaction (qRT-PCR) was solely conducted on the characteristic target proteins of IM-FT. Secondly, the relatively modest sample size may restrict the generalizability of our analytical findings and compromise statistical power. Furthermore, the absence of thorough clinical validation analyses undermines the clinical translational potential of the results. Finally, the integration of multiple datasets may have introduced potential batch effects, which could interfere with the stability and reliability of the observed gene expression patterns. These limitations highlight the imperative of enhancing experimental validation and expanding cohort sizes in future research to ensure the robustness of conclusions.

Notwithstanding these limitations, our integrated bioinformatics and experimental investigation offers a comprehensive insight into the molecular mechanisms underlying NAFLD pathogenesis. The identified biomarkers exhibit robust diagnostic performance, whereas the defined molecular subtypes display distinct immunological and functional features. Additionally, we provide valuable clues regarding potential therapeutic agents targeting IM-FT. Collectively, these findings substantially advance the current understanding of NAFLD pathogenesis and establish a solid foundation for future research and clinical applications aimed at improving the prevention, diagnosis, and treatment of this prevalent and debilitating disease.

## Data Availability

The datasets presented in this study can be found in online repositories. The names of the repository/repositories and accession number(s) can be found in the article/Supplementary Material.
