# Supplementary material for: SLC11A1 protein as a key regulator of iron metabolism, ferroptosis mediator, and putative therapeutic target in nonalcoholic fatty liver disease: an integrated bioinformatics analysis
Source: Front Pharmacol. 2025 Nov 25;16:1715699. doi: 10.3389/fphar.2025.1715699 (PMC12685672; doi:10.3389/fphar.2025.1715699)
Supplement: Supplementary file 1 [file Supplementaryfile1.zip › Supplementary Material/Mold-making method.docx]

The high-fat diet (HFD) model involves feeding mice or rats a high-fat diet (typically containing 45%–60% fat) for 8–24 weeks, which induces obesity, insulin resistance, and hepatic steatosis. As the most widely used model, it faithfully recapitulates the metabolic characteristics of metabolic dysfunction-associated fatty liver disease (MAFLD) in humans.

[1]Kucera O, Cervinkova Z. Experimental models of non-alcoholic fatty liver disease in rats. World J Gastroenterol. 2014 Jul 14;20(26):8364-76. doi: 10.3748/wjg.v20.i26.8364

[2]Santhekadur PK, Kumar DP, Sanyal AJ. Preclinical models of non-alcoholic fatty liver disease. J Hepatol. 2018 Feb;68(2):230-237. doi: 10.1016/j.jhep.2017.10.031

[3]Riordan JD, Nadeau JH. Modeling progressive non-alcoholic fatty liver disease in the laboratory mouse. Mamm Genome. 2014 Oct;25(9-10):473-86. doi: 10.1007/s00335-014-9521-3
