# Supplementary figures and images for: SLC11A1 protein as a key regulator of iron metabolism, ferroptosis mediator, and putative therapeutic target in nonalcoholic fatty liver disease: an integrated bioinformatics analysis
Source: Front Pharmacol. 2025 Nov 25;16:1715699. doi: 10.3389/fphar.2025.1715699 (PMC12685672; doi:10.3389/fphar.2025.1715699)

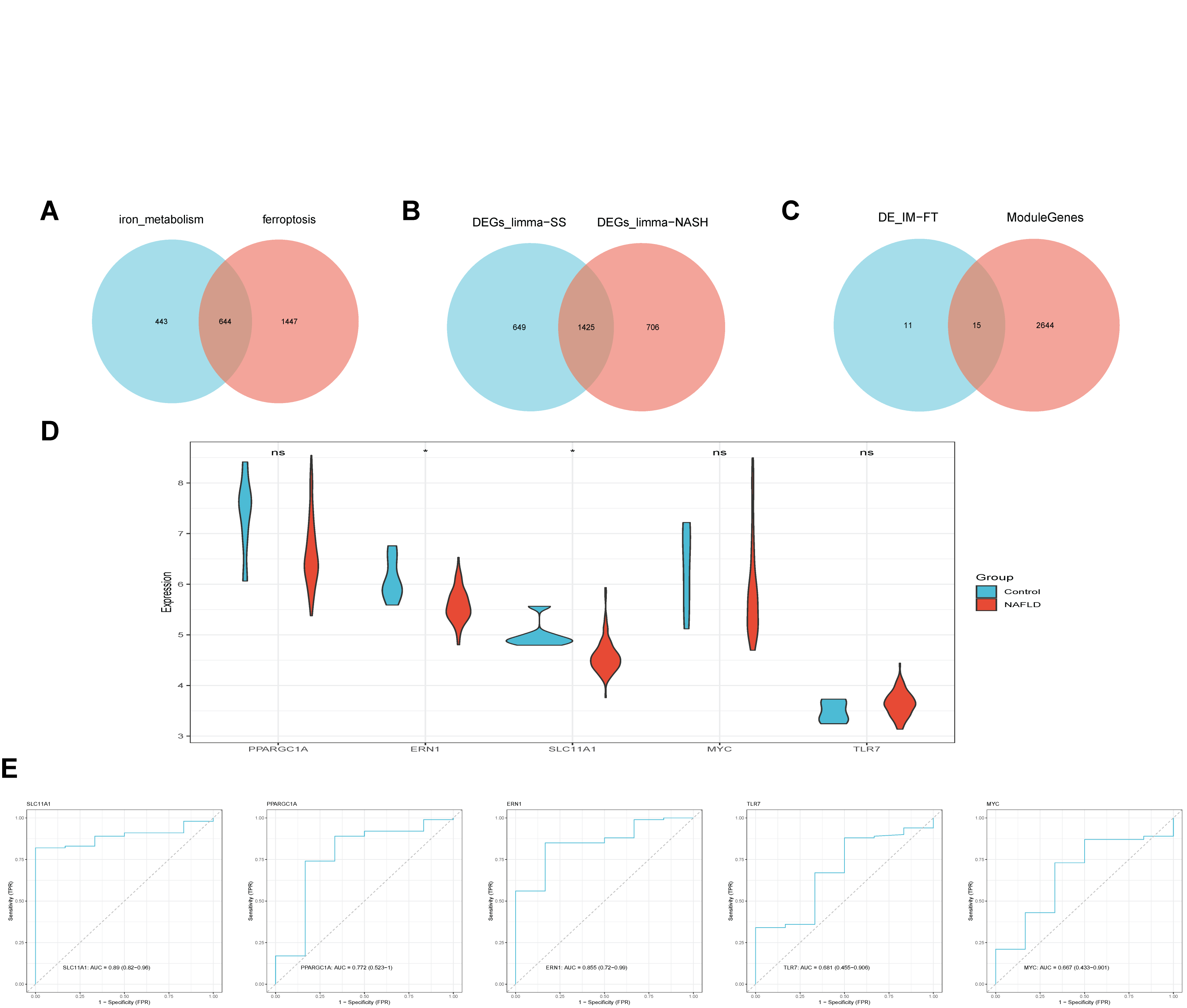

Supplement: Supplementary file 1 [file Supplementaryfile1.zip › Supplementary Material/Supplementary Fiugre/figS.tif]

iron\_metabolism

ferroptosis

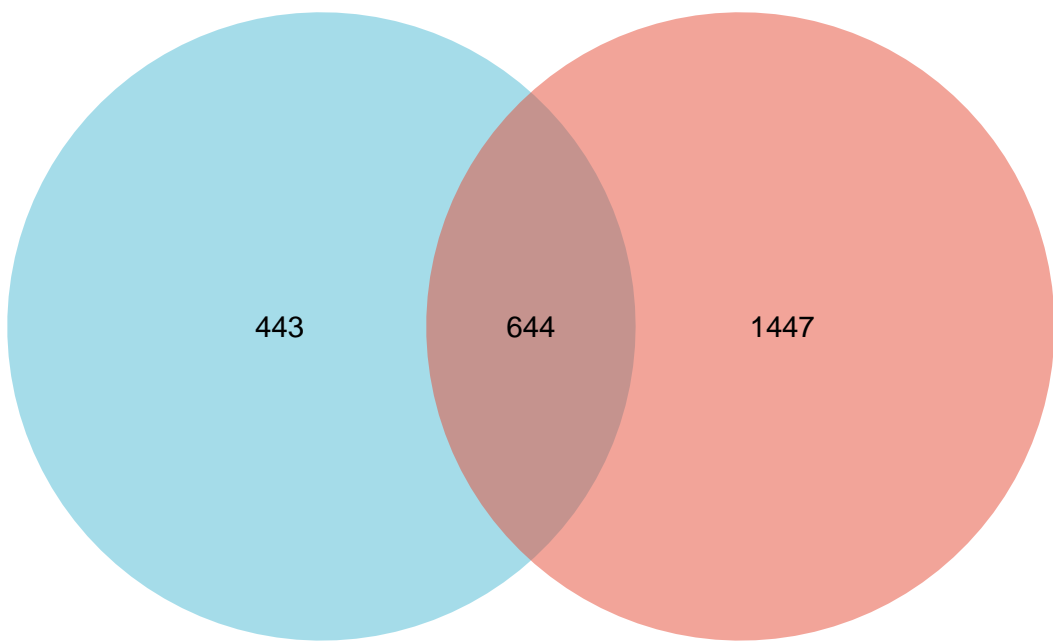

Supplement: Supplementary file 1 [file Supplementaryfile1.zip › Supplementary Material/Supplementary Fiugre/SA.pdf]

DEGs\_limma-SS

DEGs\_limma-NASH

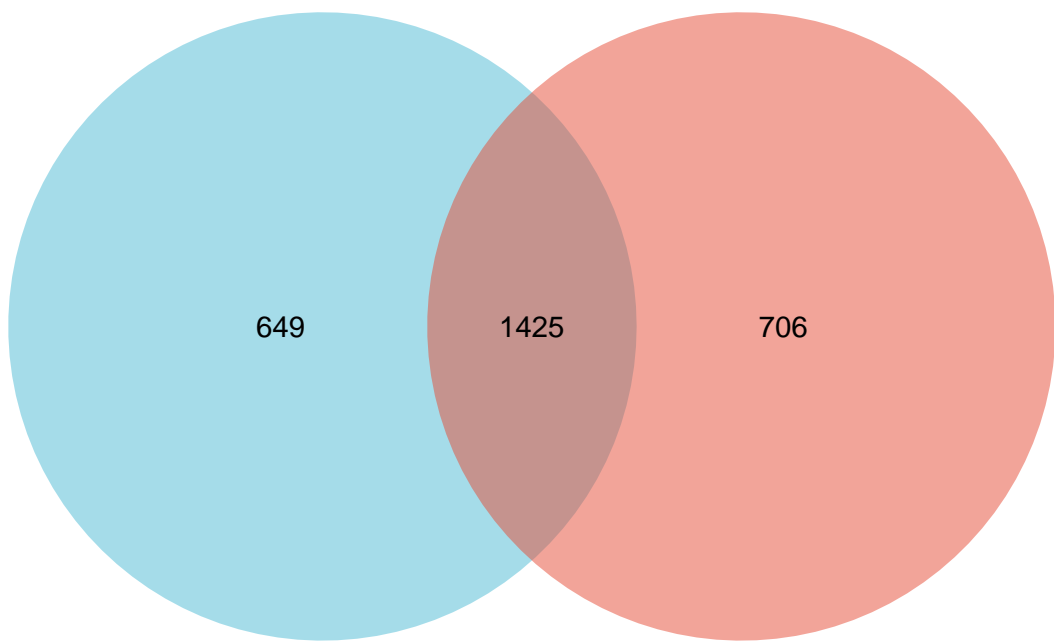

Supplement: Supplementary file 1 [file Supplementaryfile1.zip › Supplementary Material/Supplementary Fiugre/SB.pdf]

DE\_IM-FT

ModuleGenes

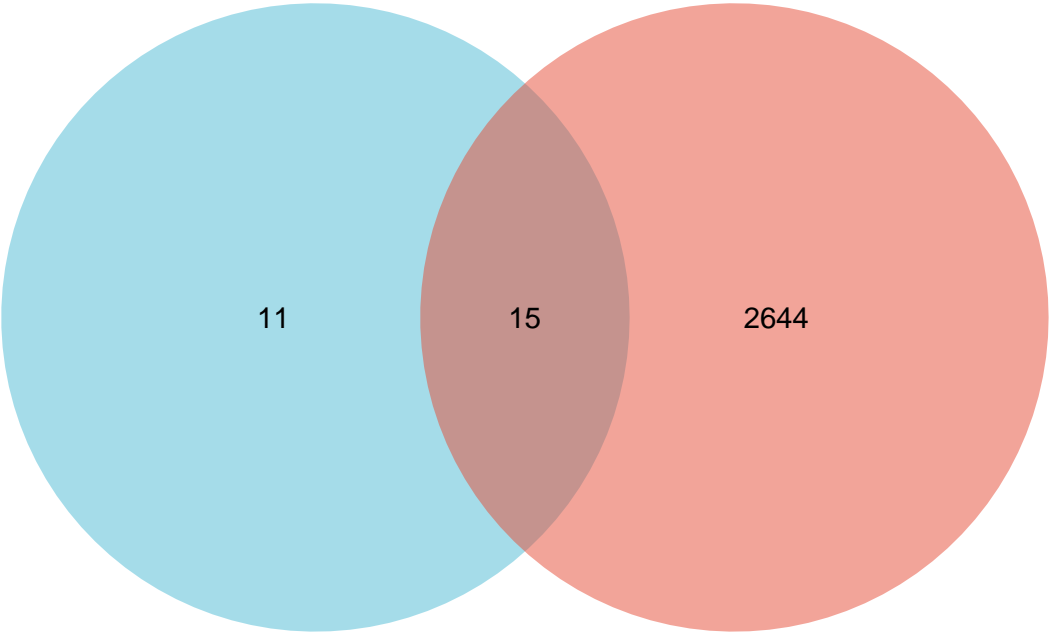

Supplement: Supplementary file 1 [file Supplementaryfile1.zip › Supplementary Material/Supplementary Fiugre/SC.pdf]

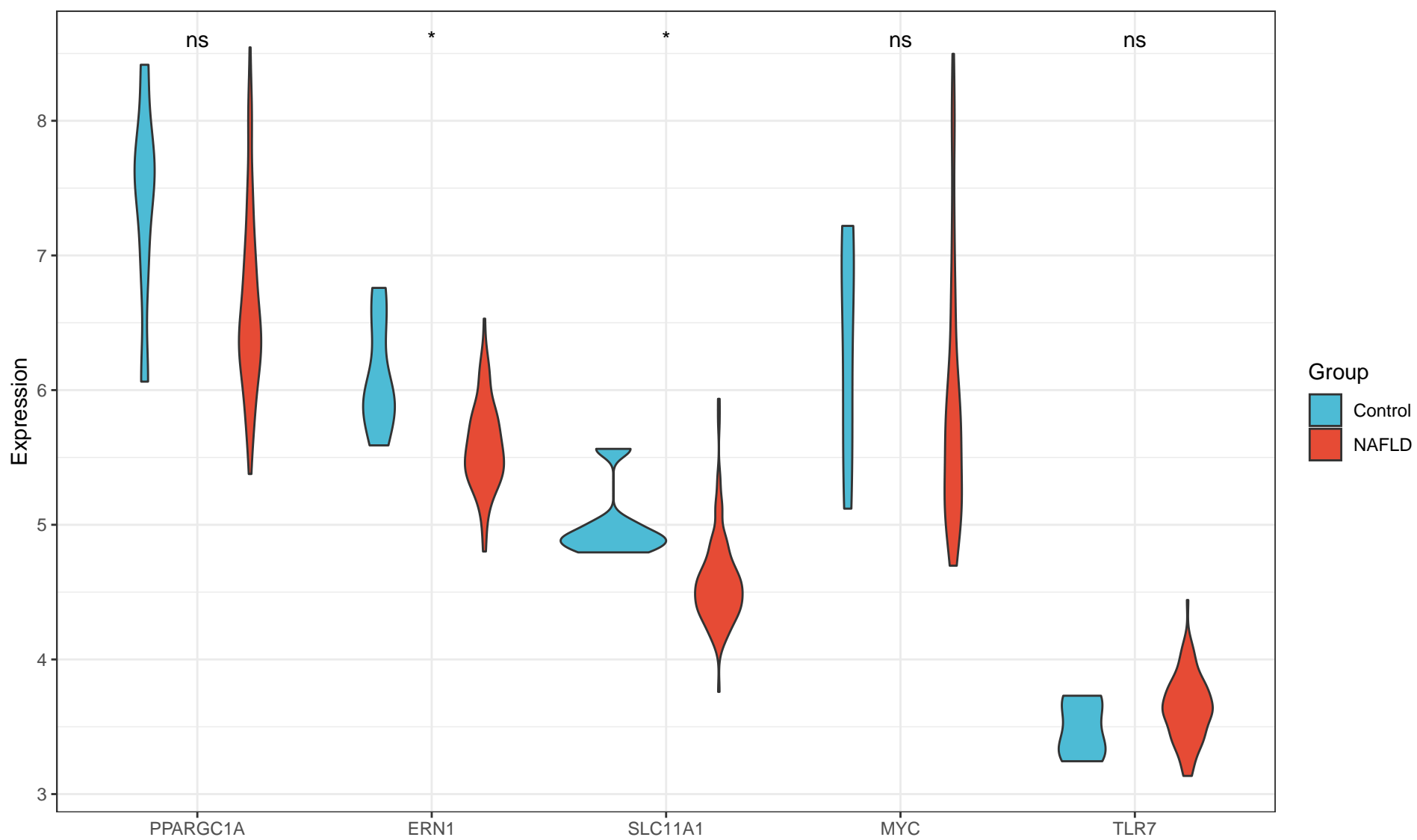

Supplement: Supplementary file 1 [file Supplementaryfile1.zip › Supplementary Material/Supplementary Fiugre/SD.pdf]

ERN1

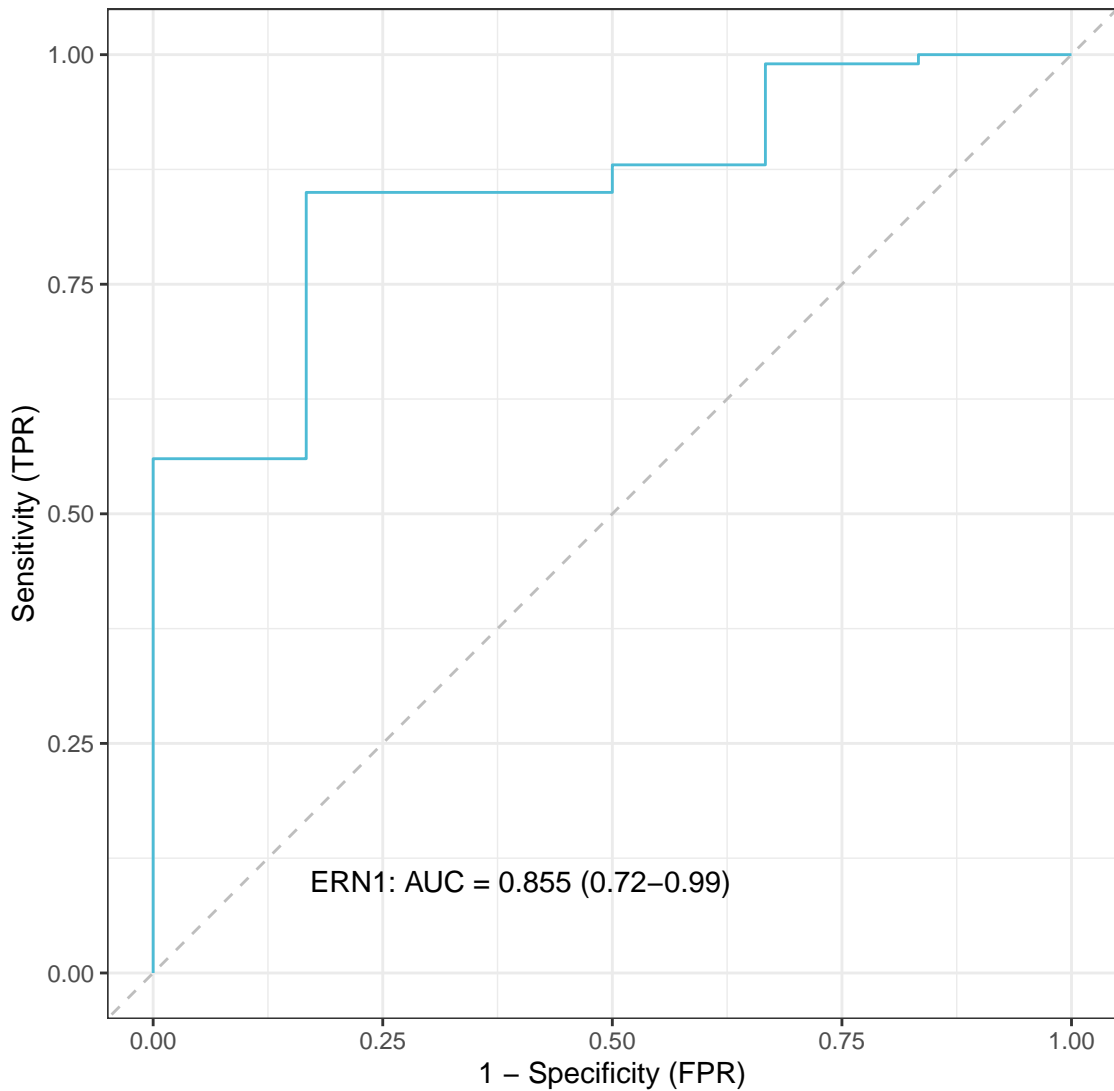

Supplement: Supplementary file 1 [file Supplementaryfile1.zip › Supplementary Material/Supplementary Fiugre/SE-ERN1.pdf]

MYC

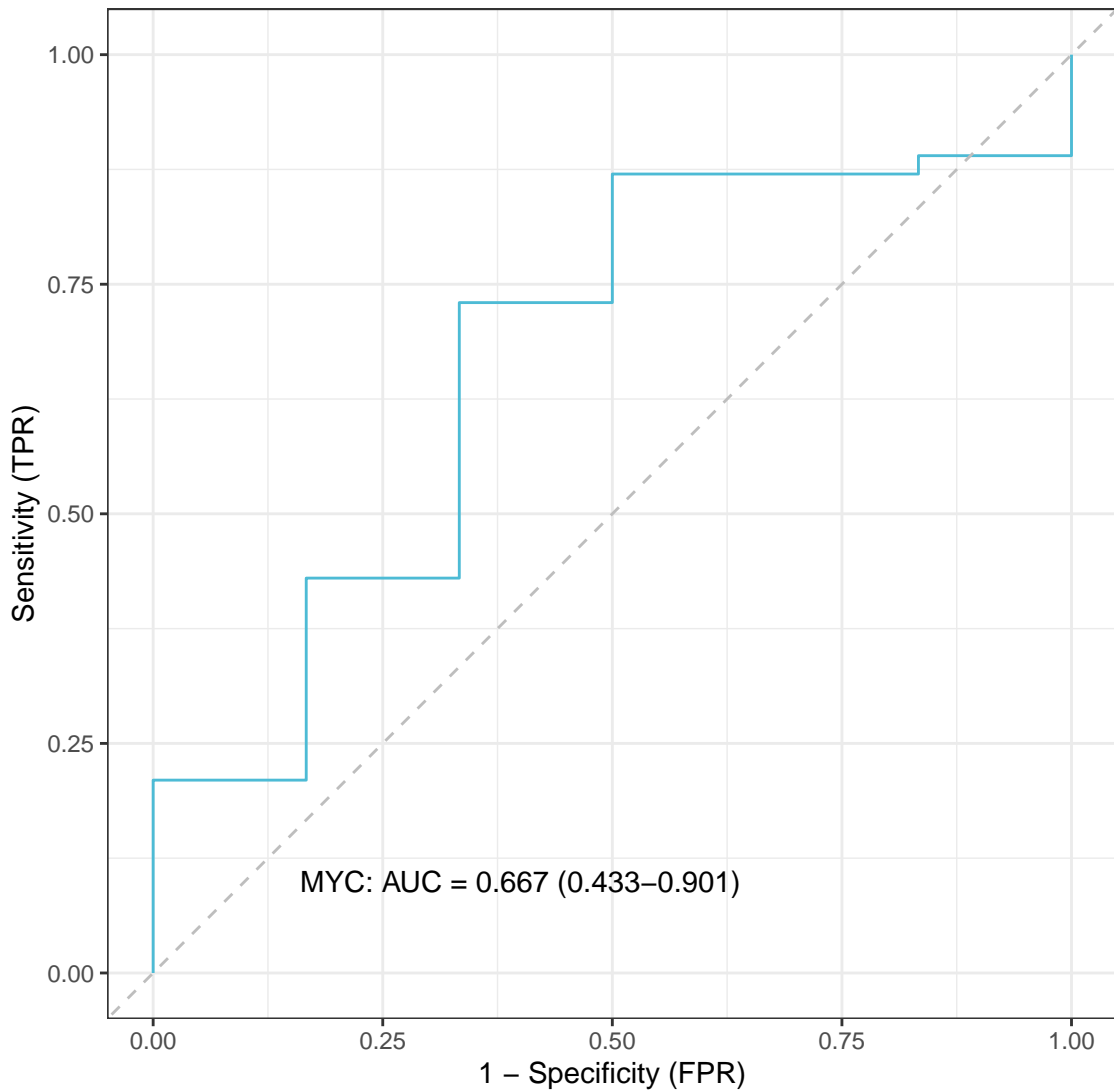

Supplement: Supplementary file 1 [file Supplementaryfile1.zip › Supplementary Material/Supplementary Fiugre/SE-MYC.pdf]

PPARGC1A

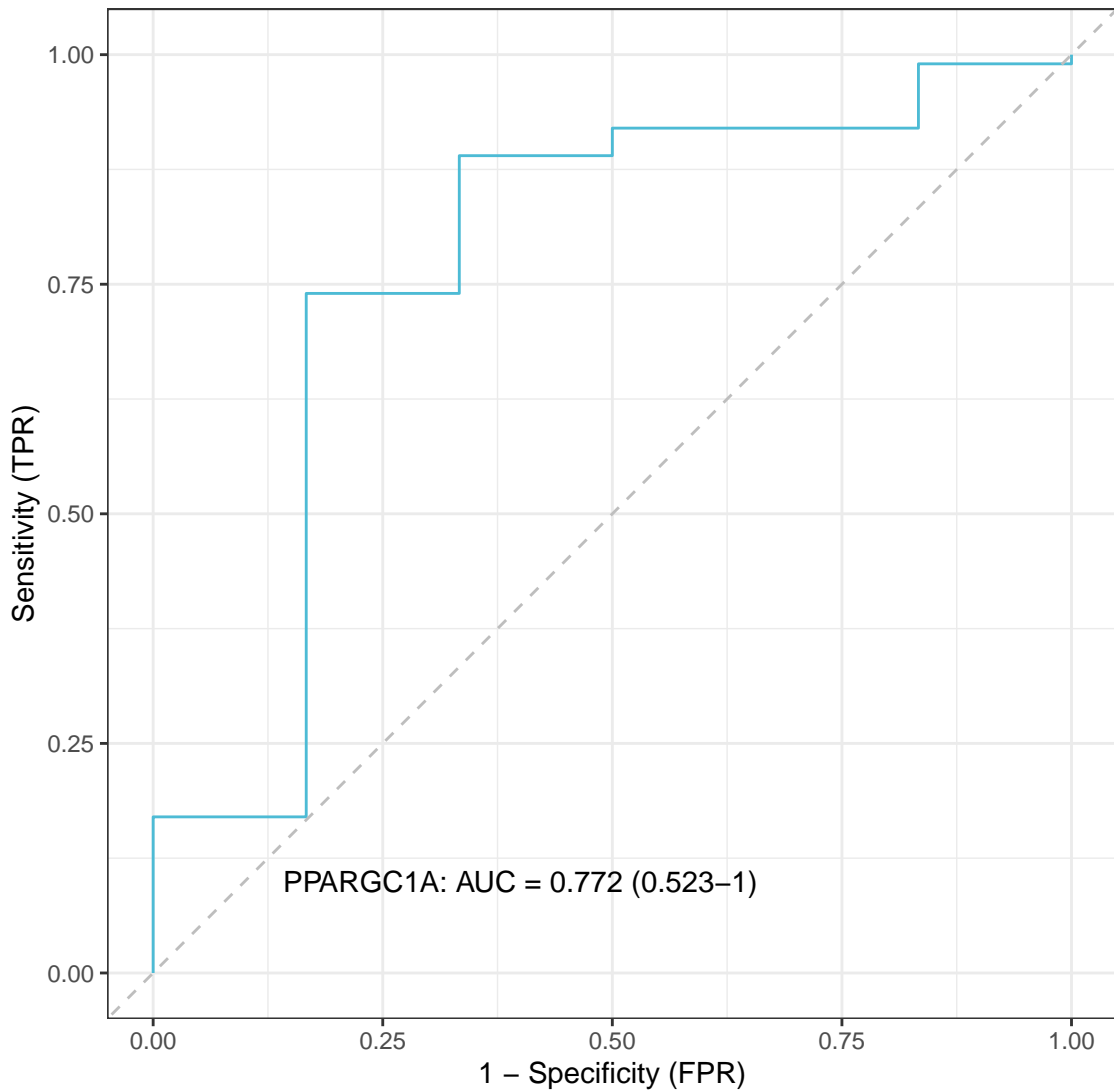

Supplement: Supplementary file 1 [file Supplementaryfile1.zip › Supplementary Material/Supplementary Fiugre/SE-PPARGC1A.pdf]

SLC11A1

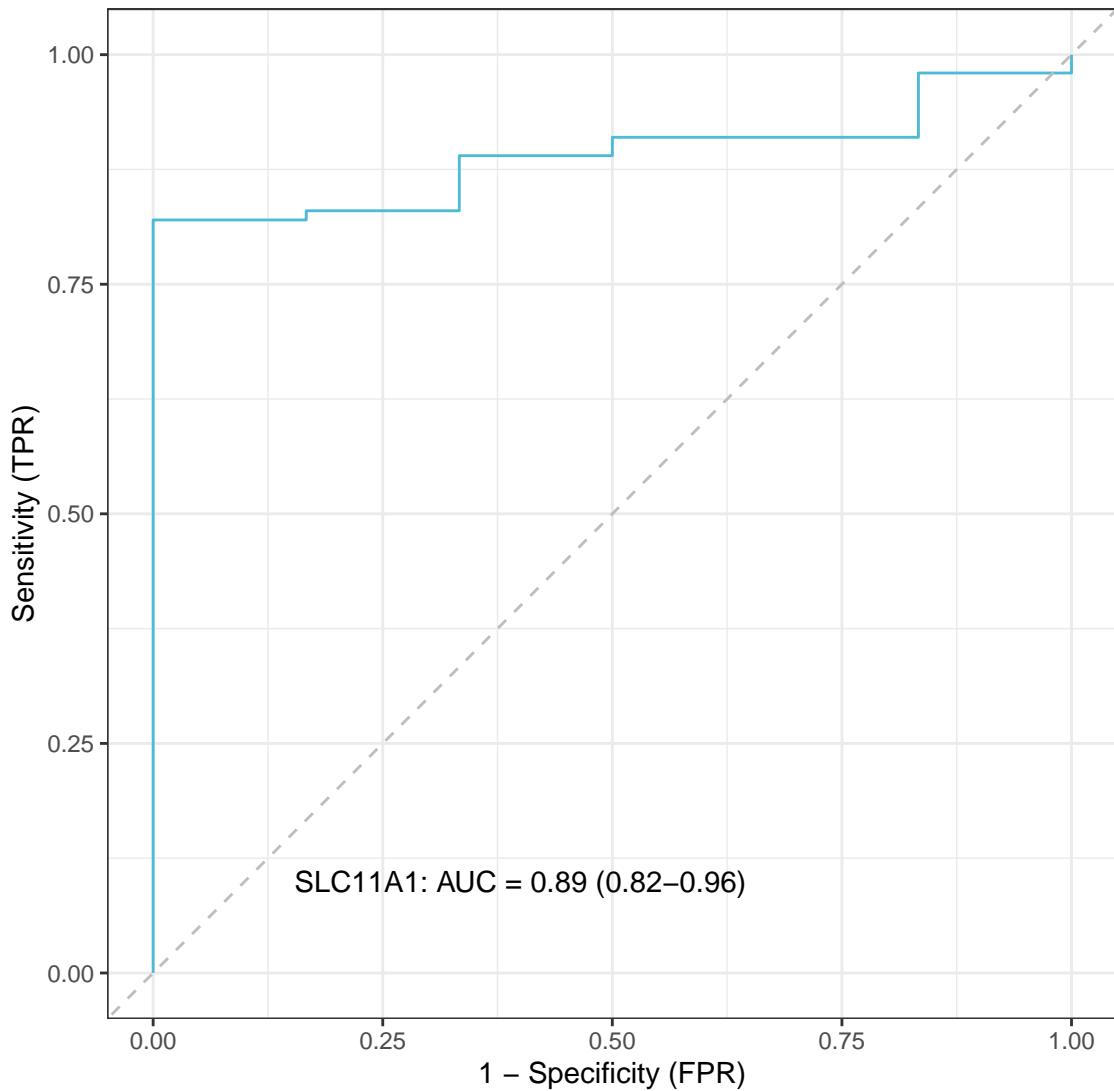

Supplement: Supplementary file 1 [file Supplementaryfile1.zip › Supplementary Material/Supplementary Fiugre/SE-SLC11A1.pdf]

TLR7

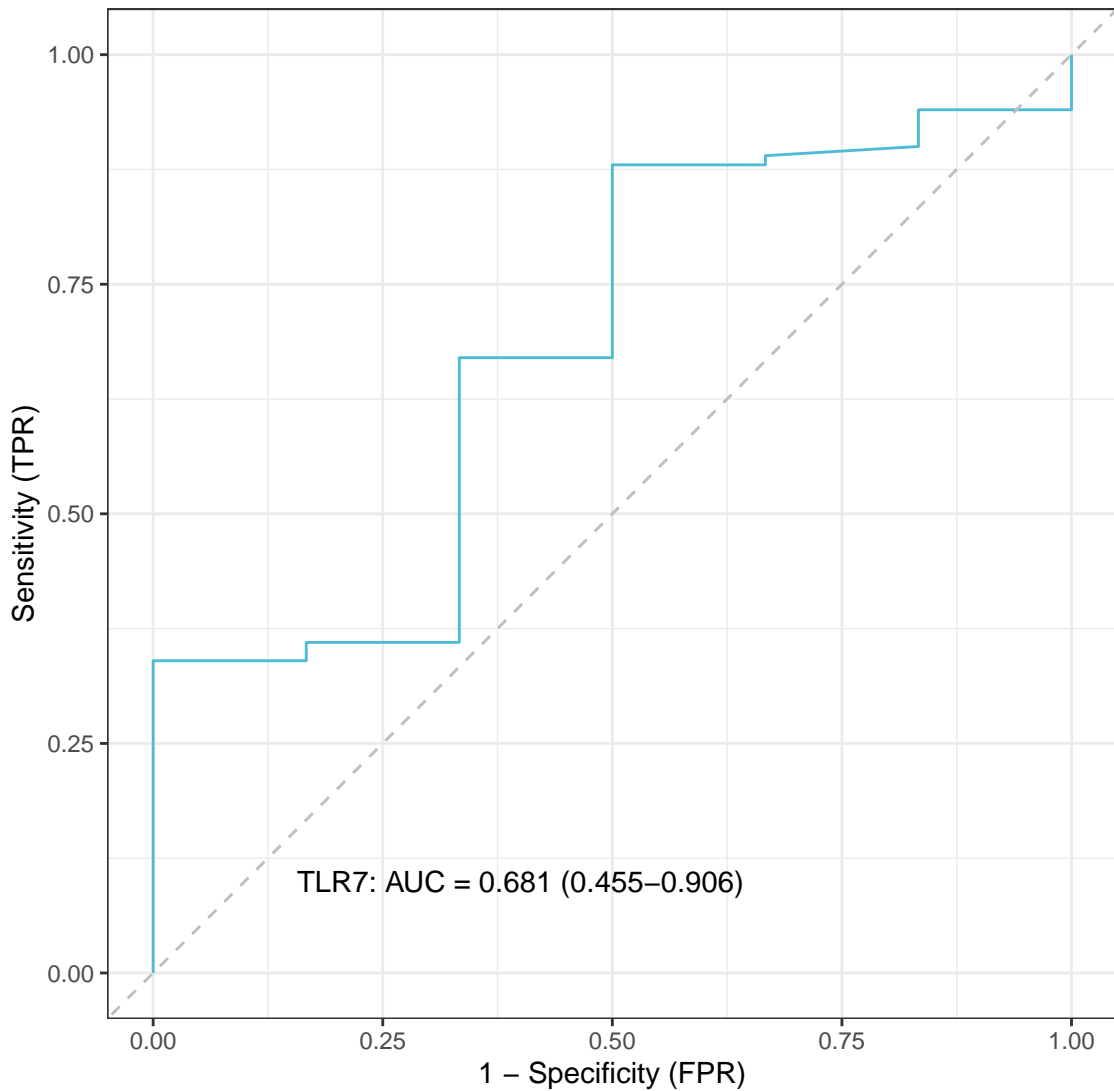

Supplement: Supplementary file 1 [file Supplementaryfile1.zip › Supplementary Material/Supplementary Fiugre/SE-TLR7.pdf]
